# Single-Domain Antibodies for Targeting, Detection, and *In Vivo* Imaging of Human CD4^+^ Cells

**DOI:** 10.3389/fimmu.2021.799910

**Published:** 2021-12-09

**Authors:** Bjoern Traenkle, Philipp D. Kaiser, Stefania Pezzana, Jennifer Richardson, Marius Gramlich, Teresa R. Wagner, Dominik Seyfried, Melissa Weldle, Stefanie Holz, Yana Parfyonova, Stefan Nueske, Armin M. Scholz, Anne Zeck, Meike Jakobi, Nicole Schneiderhan-Marra, Martin Schaller, Andreas Maurer, Cécile Gouttefangeas, Manfred Kneilling, Bernd J. Pichler, Dominik Sonanini, Ulrich Rothbauer

**Affiliations:** ^1^ NMI Natural and Medical Sciences Institute at the University of Tübingen, Reutlingen, Germany; ^2^ Werner Siemens Imaging Center, Department of Preclinical Imaging and Radiopharmacy, University of Tübingen, Tübingen, Germany; ^3^ Department of Immunology, Institute of Cell Biology, University of Tübingen, Tübingen, Germany; ^4^ Pharmaceutical Biotechnology, University of Tübingen, Tübingen, Germany; ^5^ German Cancer Consortium (DKTK) and German Cancer Research Center (DKFZ) partner site Tübingen, Tübingen, Germany; ^6^ Livestock Center of the Faculty of Veterinary Medicine, Ludwig Maximilians University Munich, Oberschleissheim, Germany; ^7^ Department of Dermatology, University of Tübingen, Tübingen, Germany; ^8^ Cluster of Excellence iFIT (EXC2180) “Image-Guided and Functionally Instructed Tumor Therapies,” University of Tübingen, Tübingen, Germany; ^9^ Department of Medical Oncology and Pneumology, University of Tübingen, Tübingen, Germany

**Keywords:** CD4, nanobody, immune tracer, PET imaging, magnetic resonance imaging, immunotherapies

## Abstract

The advancement of new immunotherapies necessitates appropriate probes to monitor the presence and distribution of distinct immune cell populations. Considering the key role of CD4^+^ cells in regulating immunological processes, we generated novel single-domain antibodies [nanobodies (Nbs)] that specifically recognize human CD4. After in-depth analysis of their binding properties, recognized epitopes, and effects on T-cell proliferation, activation, and cytokine release, we selected CD4-specific Nbs that did not interfere with crucial T-cell processes *in vitro* and converted them into immune tracers for noninvasive molecular imaging. By optical imaging, we demonstrated the ability of a high-affinity CD4-Nb to specifically visualize CD4^+^ cells *in vivo* using a xenograft model. Furthermore, quantitative high-resolution immune positron emission tomography (immunoPET)/MR of a human CD4 knock-in mouse model showed rapid accumulation of ^64^Cu-radiolabeled CD4-Nb1 in CD4^+^ T cell-rich tissues. We propose that the CD4-Nbs presented here could serve as versatile probes for stratifying patients and monitoring individual immune responses during personalized immunotherapy in both cancer and inflammatory diseases.

## Introduction

In precision medicine, diagnostic classification of the disease-associated immune status should guide the selection of appropriate therapies. A comprehensive analysis of a patient’s specific immune cell composition, activation state, and infiltration of affected tissue has been shown to be highly informative for patient stratification (1–3). In this context, CD4 is an important marker, as it is found on the surface of immune cells such as monocytes, macrophages, and dendritic cells and most abundant on CD4^+^ T cells (4, 5). CD4^+^ T cells are a key determinant of the immune status due to their essential role in orchestrating immune responses in autoimmune diseases, immune-mediated inflammatory diseases (IMIDs), cancer, and chronic viral infections (6–13). Current diagnostic standards such as intra-cytoplasmic flow cytometry analysis (IC-FACS), immunohistochemistry, and *ex vivo* cytokine assays or RT-PCR analysis are exclusively invasive and therefore limited with respect to repetitive analyses over time (14–17). Considering the emerging role of infiltrating lymphocytes and the impact of CD4^+^ T cells on the outcome of immunotherapies, novel approaches are needed to assess CD4^+^ T cells more holistically (18). In this context, noninvasive imaging approaches offer a significant benefit compared to the current diagnostic standard. To date, radiolabeled antibodies have been applied to image CD4^+^ cells in preclinical models (10, 19–21). Due to the recycling effect mediated by the neonatal Fc receptor, full-length antibodies have a long serum half-life, which requires long clearance times of several days before high-contrast images can be acquired (22). Additionally, effector function *via* the Fc region was shown to induce depletion or functional changes in CD4^+^ cells including the induction of proliferation or cytokine release (23–25). Notably, also higher dosages of recombinant antibody fragments like Fab fragments or Cys-diabodies derived from the monoclonal anti-CD4 antibody GK1.5 were recently shown to decrease CD4 expression *in vivo* and inhibit proliferation and interferon (IFN)-γ production *in vitro* (24–26). These studies highlight the importance of carefully investigating CD4^+^ cell-specific immunoprobes for their epitopes, binding properties, and functional effects.

During the last decade, antibody fragments derived from heavy-chain-only antibodies of camelids, referred to as VHHs or nanobodies (Nbs) (27), have emerged as versatile probes for molecular imaging [reviewed in (28)]. In combination with highly sensitive and/or quantitative whole-body molecular imaging techniques such as optical or radionuclide-based modalities, particularly positron emission tomography (PET), Nbs have been shown to bind their targets within several minutes of systemic application (29). Due to their great potential as highly specific imaging probes, numerous Nbs targeting immune- or tumor-specific cellular antigens are currently in preclinical development and even in clinical trials (28, 30, 31).

Here, we generated a set of human CD4 (hCD4)-specific Nbs. Following in-depth characterization of their binding properties, we selected candidates that did not affect T-cell proliferation, activation, or cytokine release and converted them into immune tracers for noninvasive optical and PET imaging. Using a mouse xenograft model and an hCD4 knock-in mouse model, we successfully demonstrated the capacity of these CD4-Nbs to visualize CD4^+^ cells *in vivo*.

## Results

### Generation of High-Affinity CD4 Nanobodies

**Figure 1:**
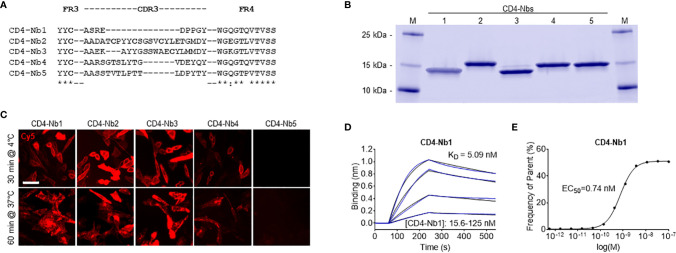
Identification and characterization of nanobodies (Nbs) against human CD4 (hCD4). **(A)** Amino acid sequences of the complementarity determining region (CDR) 3 from unique CD4-Nbs selected after two rounds of biopanning are listed. **(B)** Recombinant expression and purification of CD4-Nbs using immobilized metal ion affinity chromatography (IMAC) and size exclusion chromatography (SEC). Coomassie-stained SDS-PAGE of 2 µg of purified Nbs is shown. **(C)** Representative images of live CHO-hCD4 cells stained with CD4-Nbs for 30 min at 4°C (top row) or 60 min at 37°C (bottom row); scale bar: 50 µm. **(D)** For biolayer interferometry (BLI)-based affinity measurements, biotinylated hCD4 was immobilized on streptavidin biosensors. Kinetic measurements were performed using four concentrations of purified Nbs ranging from 15.6 to 1,000 nM. As an example, the sensogram of CD4-Nb1 at indicated concentrations is shown. **(E)** EC_50_ determination by flow cytometry. Exemplarily shown for CD4-Nb1, the percentage of positively stained HEK293-hCD4 (frequency of parent) was plotted against indicated concentrations of CD4-Nbs.

To generate Nbs directed against hCD4, we immunized an alpaca (*Vicugna pacos*) with the recombinant extracellular portion of hCD4 following an 87-day immunization protocol. Subsequently, we generated a Nb phagemid library comprising ~4 × 10^7^ clones that represent the full repertoire of variable heavy chains of heavy-chain antibodies (VHHs or Nbs) of the animal. We performed phage display using either passively adsorbed purified hCD4 or CHO and HEK293 cells stably expressing full-length hCD4 (CHO-hCD4 and HEK293-hCD4 cell lines, respectively). Following two cycles of phage display for each condition, we analyzed a total of 612 individual clones by whole-cell phage ELISA and identified 78 positive binders. Sequence analysis revealed 13 unique Nbs representing five different B-cell lineages according to their complementarity determining region (CDR) 3 (**Figure 1A**). One representative Nb of each lineage, termed CD4-Nb1–CD4-Nb5, was expressed in bacteria (*Escherichia coli)* and isolated with high purity using immobilized metal ion affinity chromatography (IMAC) followed by size exclusion chromatography (SEC) (**Figure 1B**). To test whether selected Nbs are capable of binding to full-length hCD4 localized at the plasma membrane of mammalian cells, we performed live-cell staining of CHO-hCD4 cells (**Figure 1C**, **Supplementary Figure 1**). Executed at 4°C, images showed a prominent staining of the plasma membrane, whereas at 37°C, the fluorescent signal was mainly localized throughout the cell body, presumably a consequence of endocytotic uptake of receptor-bound Nbs. CHO wild-type (wt) cells were not stained by any of the five CD4-Nbs at both temperatures (data not shown). CD4-Nb1 and CD4-Nb3, both identified by whole-cell panning, displayed strong staining of CHO-hCD4 cells. Of the Nbs derived from panning with recombinant hCD4, CD4-Nb2 also showed strong cellular staining, whereas staining with CD4-Nb4 revealed weak signals. CD4-Nb5 showed no staining under these conditions and was consequently excluded from further analyses (**Figure 1C**). To quantitatively assess binding affinities, we performed biolayer interferometry (BLI), measuring serial dilutions of Nbs on the biotinylated extracellular domain of hCD4 immobilized at the sensor tip. For CD4-Nb1 and CD4-Nb2, K_D_ values were determined to be ~5 and ~7 nM, respectively, while CD4-Nb3 and CD4-Nb4 displayed lower affinities of 75 and 135 nM, respectively (**Figure 1D**, **Table 1**, **Supplementary Figure 2A**). In addition, we determined corresponding EC_50_ values with full-length plasma membrane-located hCD4 on HEK293-hCD4 cells by flow cytometry. In accordance with cellular staining and biochemically determined affinities, these values revealed a strong functional binding for CD4-Nb1 and CD4-Nb2 with EC_50_ values in the subnanomolar range (~0.7 nM), whereas CD4-Nb3 and CD4-Nb4 displayed substantially lower cellular affinities (**Figure 1E**, **Table 1**, **Supplementary Figure 2B**). In summary, we generated four CD4-Nbs that bind isolated and cell-resident hCD4. While CD4-Nb3 and CD4-Nb4 appeared less affine, CD4-Nb1 and CD4-Nb2 displayed high affinities in the low nanomolar range.

**Table 1:** Summary of affinities (k_D_) and association (k_on_) and dissociation constants (k_off_ and coefficient of determination R_2_) determined by BLI (left side) and EC_50_ values of flow cytometry (right side).

	Dissociation constant K_D_	k_on_ (10^5^ M^-1^ s^-1^)	k_off_ (10^-4^ s^-1^)	R^2^	EC_50_
CD4-Nb1	5.1 nM	1.21 ± 0.022	6.13 ± 0.27	0.996	0.74 nM
CD4-Nb2	6.5 nM	1.22 ± 0.015	7.95 ± 0.18	0.998	0.73 nM
CD4-Nb3	75.3 nM	0.82 ± 0.026	61.8 ± 2.00	0.983	533 nM
CD4-Nb4	135 nM	1.18 ± 0.014	160 ± 0.97	0.998	7.36 µM

### Domain Mapping

**Figure 2:**
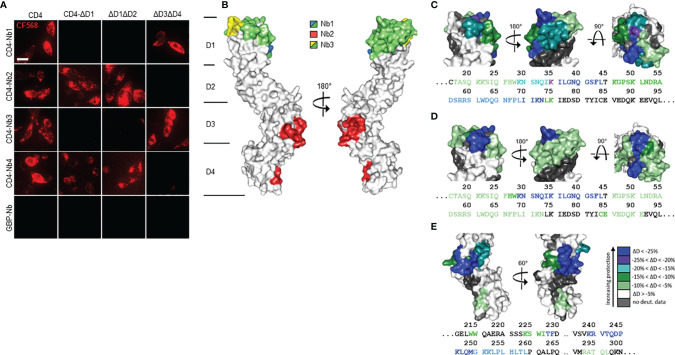
Localization of CD4-nanobody (Nb) binding epitopes. **(A)** Representative images of live CHO cells expressing full-length or domain-deletion mutants of human CD4 (hCD4) stained with fluorescently labeled CD4-Nbs (CF568) are shown; scale bar 10 µm. **(B)** Surface structure model of hCD4 (PDBe 1wiq) (36) and the hydrogen-deuterium exchange mass spectrometry (HDX-MS) epitope mapping results of CD4-Nb1–3 are depicted. Different colors highlight the amino acid residues protected by CD4-Nb1 (blue), CD4-Nb2 (red), or CD4-Nb3 (yellow). Overlapping residues protected by both Nb1 and Nb3 are colored green. A more detailed surface map (%ΔD) of these specific regions is highlighted in **(C)** (CD4-Nb1), **(D)** (CD4-Nb3), and **(E)** (CD4-Nb2) with the corresponding CD4 amino acid sequence.

Next, we applied chemo-enzymatic coupling using sortase A for site-directed functionalization of CD4-Nbs (32, 33). We thereby linked peptides conjugated to a single fluorophore to the C-terminus of CD4-Nbs, yielding a defined labeling ratio of 1:1 (34). Live-cell immunofluorescence imaging showed that all sortase-coupled CD4-Nbs retained their capability of binding to cell-resident hCD4 of CHO-hCD4 cells (**Supplementary Figure 3A**). To localize the binding sites of the selected CD4-Nbs, we generated domain-deletion mutants of hCD4. Expression and correct surface localization of these mutants in CHO cells were confirmed by staining with antibody RPA-T4 binding to domain 1 of CD4. For mutants lacking domain 1, we introduced an N-terminal BC2 tag (35) to allow for live-cell surface detection with a fluorescently labeled bivBC2-Nb (34) (**Supplementary Figure 3B**). Transiently expressed domain-deletion mutants were then tested for binding of CF568-labeled CD4-Nbs by live-cell immunofluorescence imaging, including a non-specific fluorescently labeled green fluorescent protein (GFP)-binding Nb (GFP-Nb) as negative control. Based on these results, we allocated binding of CD4-Nb1 and CD4-Nb3 to domain 1, whereas CD4-Nb2 and CD4-Nb4 bind to domain 3 and/or 4 of hCD4 (**Figure 2A**, **Supplementary Figure 3C**).

To further examine combinatorial binding of the different CD4-Nbs, we performed an epitope binning analysis by BLI. Recombinant full-length hCD4 was immobilized at the sensor tip, and combinations of CD4-Nbs were allowed to bind consecutively (**Supplementary Figure 4**). Unsurprisingly, CD4-Nbs binding to different domains displayed combinatorial binding. Interestingly, a simultaneous binding was also detected for the combination of CD4-Nb1 and CD4-Nb3, suggesting that both CD4-Nbs bind to different epitopes within domain 1. In contrast, we did not observe simultaneous binding for CD4-Nb2 and CD4-Nb4, which might be due to close-by or overlapping epitopes at domain 3/4 for the latter Nb pair.

For a more precise epitope analysis, we conducted a hydrogen–deuterium exchange mass spectrometry (HDX-MS) analysis of hCD4 bound to CD4-Nb1, CD4-Nb2, or CD4-Nb3 (**Figures 2B–E**, **Supplementary Figure 5**). Due to its low affinity, CD4-Nb4 was not considered for HDX-MS analysis (data not shown). In accordance with our previous findings, binding of CD4-Nb1 and CD4-Nb3 protected sequences of domain 1 from HDX, whereas CD4-Nb2 protected sequences of domains 3 and 4 of hCD4 (**Figure 2B**). The results obtained for binding of CD4-Nb1 (**Figure 2C**) are similar to those obtained for CD4-Nb3 (**Figure 2D**) in that binding of either Nb reduced hydrogen exchange at amino acid (aa) residues from aa T17 to N73, albeit with a different extent of protection at individual sequence segments. For CD4-Nb1, the greatest protection from HDX was observed for the sequence ranging from aa K35 to L44 corresponding to β strand C´ and C´´ of the immunoglobulin fold of domain 1 and residues aa K46–K75, comprising β strands D and E. In contrast, binding of CD4-Nb3 confers only a minor reduction in HDX within the latter sequence but additionally protects sequence aa C84–E91, which correspond to β strands G and F and their intermediate loop. For CD4-Nb2, we found protection of sequences aa W214–F229 (β strands C and C´) and aa K239–L259 (β strands C´´–E) and to a minor extent sequence aa R293–L296 as part of β strand A of domain 4 (**Figure 2E**). In summary, our HDX-MS analysis revealed that all three tested Nbs bind three dimensional epitopes within different parts of hCD4. It further provides an explanation how CD4-Nb1 and CD4-Nb3 can bind simultaneously to domain 1 of hCD4 and confirms that the epitope of CD4-Nb2 is mainly located at domain 3.

### Binding of CD4-Nbs to Human Peripheral Blood Mononuclear Cells

**Figure 3:**
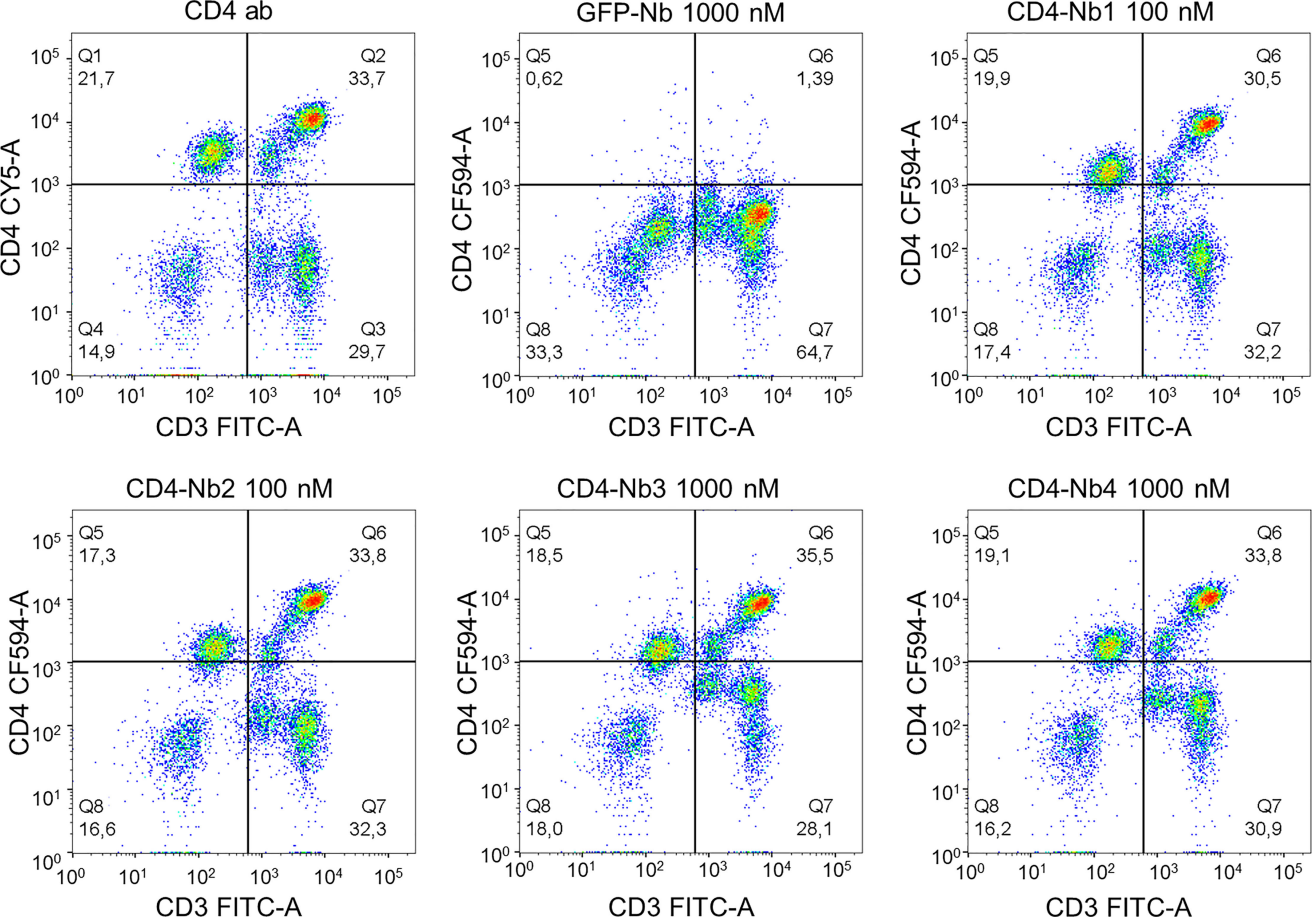
Flow cytometry analysis of human peripheral blood mononuclear cells (PBMCs) stained with fluorescently labeled CD4-nanobodies (Nbs). Schematic representation of the final gating step for CD3^+^CD4^+^ double-positive cells derived from donor 1.

Having demonstrated that all selected Nbs bind to recombinant and exogenously overexpressed cellular hCD4, we next examined their capability and specificity of binding to physiologically relevant levels of CD4^+^ T cells within peripheral blood mononuclear cell (PBMC) samples. We costained human PBMCs from three donors with CD4-Nb1–CD4-Nb4 coupled to CF568 (100 nM for high-affine CD4-Nb1 and CD4-Nb2; 1,000 nM for low-affine CD4-Nb3 and CD4-Nb4) in combination with an anti-CD3 antibody and analyzed the percentage of double-positive cells (CD3^+^CD4^+^) by flow cytometry (**Figure 3**, **Supplementary Figure 6**). Compared to staining with an anti-CD4 antibody used as a positive control, all CD4-Nbs stained a similar percentage of CD4^+^ T cells for all tested donors, while the non-specific GFP-Nb yielded a negligible percentage of double-positive cells even at the highest concentration (1,000 nM) (**Table 2**). Our analysis further revealed that, as observed with a conventional anti-CD4 antibody, the CD4-Nbs stain a substantial proportion of CD3^-^ cells, indicating that all selected candidates are also able to recognize cells such as monocytes, macrophages, or dendritic cells that express lower levels of CD4 (**Figure 3**).

**Table 2:** Percentage of double-positive cells of three donors, stained with CD4-Nb1 or CD4-Nb2 (100 nM), or CD4-Nb3 or CD4-Nb4 (1,000 nM), compared to anti-CD4 antibody and negative control Nb (GFP-Nb, 1,000 nM).

		Frequency CD3^+^ CD4^+^ (%)		
	c (nM)	Donor 1	Donor 2	Donor 3
Anti-CD4 antibody	~1	33.7	27.0	*24.3*
CD4-Nb1	100	30.5	29.2	22.7
CD4-Nb2	100	33.8	25.6	18.4
CD4-Nb3	1,000	35.5	26.5	20.4
CD4-Nb4	1,000	33.8	26.9	23.9
GFP-Nb	1,000	1.4	0.3	1.0

### Impact of CD4-Nbs on Activation, Proliferation, and Cytokine Release of CD4^+^ T and Immune Cells

In view of the envisioned application as clinical imaging tracer, we next evaluated the potential of the Nbs to be further developed into clinically approved binding molecules. Since CD4-Nb2 and CD4-Nb3 contain a number of cysteine residues in their CDR3, we excluded them at this stage because such non-canonical unpaired cysteines are often associated with expression problems and a higher tendency to form aggregates in downstream production (37, 38). With CD4-Nb1 and CD4-Nb4, we pursued two candidates that do not contain non-canonical cysteines and also cover a broad affinity spectrum. For these two Nbs and a non-specific GFP-Nb as a control, we then examined their influence on CD4^+^ T-cell activation, proliferation, and cytokine release. To rule out adverse effects of bacterial endotoxins in the Nb preparations, we first removed endotoxins by depletion chromatography, resulting in Food and Drug Administration (FDA)-acceptable endotoxin levels of <0.25 EU per mg. Typically, Nb-based radiotracers are applied at serum concentrations between 0.01 and 0.2 µM in (pre)clinical imaging (39, 40). To investigate the effects of Nbs at the expected, but also at a 10-fold increased concentration and consequently elongated serum retention times that might occur during *in vivo* (pre)clinical imaging, we treated carboxyfluorescein succinimidyl ester (CFSE)-labeled human PBMCs from three preselected healthy donors with three Nbs at concentrations ranging from 0.05 from 5 µM for 1 h at 37°C. Subsequently, cells were washed to remove Nbs and stimulated with an antigenic [cognate major histocompatibility complex (MHC)II peptides] or a non-antigenic stimulus (phytohemagglutinin, PHA-L) and analyzed after 4, 6, and 8 days by flow cytometry with the gating strategy shown in **Supplementary Figure 7A**. According to the highly similar CFSE intensity profiles observed, the total number of cell divisions was not affected by the different Nb treatments (exemplarily shown for one of three donors on day 6; **Supplementary Figure 7A**). For samples of the same donor and time point, no substantial differences in the percentage of proliferated cells were observed between mock incubation and individual Nb treatments.

**Figure 4:**
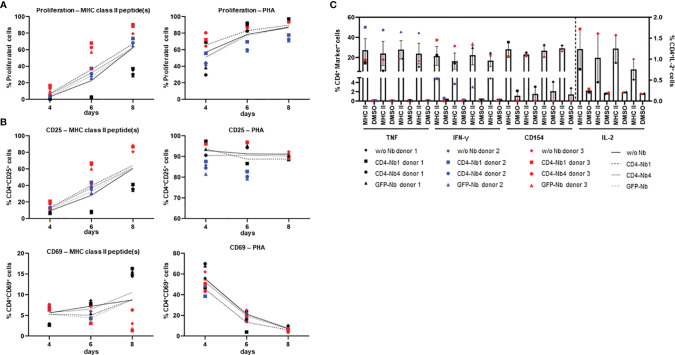
Impact of CD4-nanobodies (Nbs) on activation, proliferation, and cytokine release of T cells. Cells were stained with carboxyfluorescein succinimidyl ester (CFSE), treated with 5 µM Nbs or without for 1 h (one replicate each), washed, stimulated with 5 µg/ml MHCII peptides, 10 µg/ml PHA or not stimulated, and cultured for 12 days. **(A)** Cells were analyzed by flow cytometry for proliferation (CFSE-low/negative fraction) and activation (CD25 and CD69) on days 4, 6, and 8. Proliferation of CD4^+^ cells after stimulation with MHCII peptide(s) (left) or PHA (right). **(B)** Activation markers on CD4^+^ cells. Top: CD25 expression after stimulation with MHCII peptide(s) (left) or PHA (right); Bottom: CD69 expression after stimulation with MHCII peptide(s) (left) or PHA (right). Mean percentages of all three donors are shown as plain or dotted lines. **(C)** Cytokine and activation marker expression of CD4^+^ cells—TNF, IFN-γ, CD154 (left y-axis), or IL-2 (right y-axis). Cells were restimulated on day 12 with MHCII peptide(s) or dimethyl sulfoxide (DMSO) (background) for 14 h in the presence of Golgi Stop and brefeldin A and analyzed by flow cytometry. Error bars display SEM. Gating strategy is shown in **Supplementary Figure 6**; all percentages are given within CD4^+^ T cells.

For both stimuli, the average percentage of proliferated cells increased over time in all donors tested, with no clear differences between conditions (**Figure 4A**). As a quantitative measure of T-cell activation, we also determined the cell surface induction of a very early activation marker (CD69) and of the interleukin (IL)-2 receptor α chain (CD25) on CD4^+^ T cells (**Figure 4B**). Among samples of the same donor and stimulation, we found highly similar activation profiles for all Nb treatments. While the percentage of CD4^+^CD25^+^ cells steadily increased over time for MHCII peptide stimulation, for the PHA-stimulated condition, the percentage of positive cells was similarly high at all times of analysis. Importantly, regardless of the differences between donors, the individual Nb treatments from the same donor did not result in significant differences in the percentage of CD4^+^CD25^+^ or CD4^+^CD69^+^ cells for either stimulation at any point in the analysis.

Next, we analyzed cytokine expression of CD4^+^ T cells by intracellular cytokine staining after restimulation with cognate MHCII peptides. The corresponding gating strategy is shown in **Supplementary Figure 7B**. Samples of the same donor treated with different Nbs had highly similar percentages of cytokine [tumor necrosis factor (TNF), IFN-γ, or IL-2] or activation marker (CD154)-positive cells without stimulation and upon stimulation with MHCII peptides (**Figure 4C**). Overall, exposure to CD4-Nbs did not affect the proliferation, activation, or cytokine production of CD4^+^ T cells. In addition, we analyzed potential effects of CD4-Nbs on the release of cytokines from full-blood samples of three further donors. Upon stimulation with lipopolysaccharide (LPS) or PHA−L, we determined the serum concentrations with a panel of pro- and anti-inflammatory cytokines (**Supplementary Table 2**). Although there was significant inter-donor variation for some cytokines, Nb treatment did not result in significant differences in either stimulated or unstimulated samples (**Supplementary Figure 8**).

### CD4-Nbs for *In Vivo* Imaging

For optical *in vivo* imaging, we labeled CD4-Nbs with the fluorophore Cy5.5 (CD4-Nb-Cy5.5) by sortase-mediated attachment of an azide group followed by click-chemistry addition of dibenzocyclooctyne (DBCO)-Cy5.5. First, we tested potential cross-reactivity of the four Cy5.5-labeled CD4-Nbs to murine CD4^+^ lymphocytes. Notably, flow cytometric analysis showed that none of the selected CD4-Nbs bound murine CD4^+^ cells, suggesting exclusive binding to hCD4. Moreover, low-affine binding CD4-Nb4 bound neither mouse nor human CD4^+^ cells at the concentration used here (0.75 µg/ml, ~49 nM) (**Supplementary Figure 9**). Consequently, we focused on CD4-Nb1 as the most promising candidate and CD4-Nb4 as a candidate with a high off-target rate, both of which we further analyzed for their *in vivo* target specificity and dynamic distribution using a murine xenograft model.

**Figure 5:**
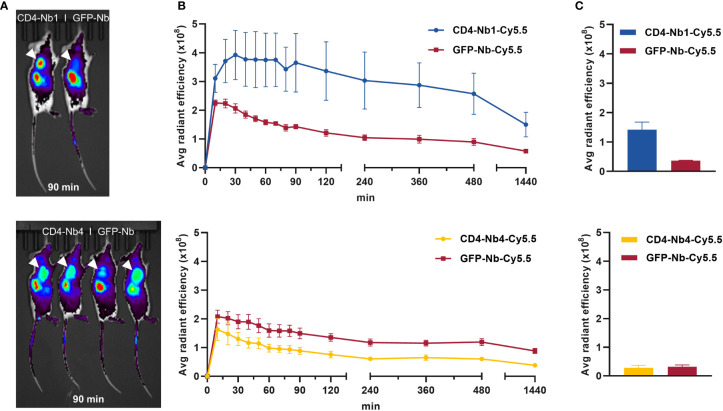
*In vivo* optical imaging (OI) with CD4-Nbs-Cy5.5. Here, 5 µg of CD4-Nbs-Cy5.5 (top) or CD4-Nb4-Cy5.5 (bottom) or GFP-Nb-Cy5.5 (top and bottom) were administered intravenously (i.v.) to subcutaneously human CD4^+^ HPB-ALL-bearing NSG mice, and tumor biodistribution was monitored by repetitive OI measurements over the course of 24h **(A)** Acquired images of each measurement time point of one representative mouse injected with CD4-Nbs-Cy5.5 (left) or GFP-Nb-Cy5.5 (right, ctrl). Red circles and white arrows indicate the tumor localization at the right upper flank. **(B)** Quantification of the fluorescence signal from the tumors (n = 4 per group, arithmetic mean of the average radiant efficiency ± SEM) determined at indicated time points. **(C)** After the last imaging time point, tumors were explanted for *ex vivo* OI, confirming increased accumulation of CD4-Nb1-Cy5.5 compared to the GFP-Nb-Cy5.5 (n = 2 per group, arithmetic mean ± SEM).

To establish hCD4-expressing tumors, NOD SCID gamma (NSG) mice were inoculated subcutaneously with CD4^+^ T-cell leukemia HPB-ALL cells (41). After 2–3 weeks, mice bearing HPB-ALL xenografts were intravenously (i.v.) injected with 5 µg of CD4-Nb1-Cy5.5, CD4-Nb4-Cy5.5, or a control Nb (GFP-Nb-Cy5.5) and non-invasively *in vivo* investigated by optical imaging (OI) in intervals over the course of 24 h (**Figure 5A**, **Supplementary Figure 10A**). The Cy5.5 signal intensity (SI) of the control Nb peaked within 10–20 minutes and rapidly declined thereafter to approximately the half and a quarter of maximum level at 2 and 24 h, respectively (**Figure 5B**, **Supplementary Figure 10B**). While the SI of the low-affinity CD4-Nb4-Cy5.5 did not exceed the SI of the control Nb at any time (**Supplementary Figure 10B**), CD4-Nb1-Cy5.5 reached its maximum SI within the HPB-ALL xenograft of ~1.8-fold above the control Nb at 30 min and slowly declined to ~90% and ~80% of maximum after 2 and 4 h, respectively (**Figure 5B**). Based on the differences in the SI between CD4-Nb1-Cy5.5 and GFP-Nb-Cy5.5, we observed constant high target accumulation and specificity between 30 and 480 min post injection (**Figure 5B**). After 24 h, mice were euthanized, and the presence of fluorophore-labeled CD4-Nbs within the explanted tumors was analyzed by OI (**Figure 5C**, **Supplementary Figure 10C**). Compared to control, tumors from mice injected with CD4−Nb1−Cy5.5 had ~4-fold higher Cy5.5 SI, indicating a good signal-to-background ratio for this Nb-derived fluorescently labeled immunoprobe even at later time points. To confirm CD4-specific targeting of CD4-Nb1 within the xenograft, we additionally performed *ex vivo* immunofluorescence of HPB-ALL tumors at 2 and 24 h post injection (**Supplementary Figure S11**). At the early time point, when the *in vivo* OI signal peaked, CD4-Nb1 was widely distributed throughout the whole tumor, whereas no Cy5.5 signal was detected in the GFP-Nb-injected mice (**Supplementary Figures 11A, B**). Semiquantitative analysis at the single-cell level revealed intense CD4-Nb1 binding at the surface of HBP-ALL cells that correlated with the CD4 antibody signal and internalization of CD4-Nb1 in some cells (**Supplementary Figure 11C**). In contrast, no binding was observed upon administration of unrelated GFP-Nb (**Supplementary Figure 11D**). At 24 h post injection, we observed regions of strongly internalized CD4-Nb1 (**Supplementary Figures 11E, G**), but also regions showing a low residual CD4-Nb1 uptake (**Supplementary Figures 11E, H**).

The OI data from the xenograft model clearly indicates that the high-affinity CD4-Nb1 but not CD4-Nb4 is suitable to specifically visualize CD4^+^ cells *in vivo* within a short period (30–120 min) after administration. Considering that this model does not reflect the natural distribution of CD4^+^ T cells in an organism, we continued with a model that allowed us to visualize the physiological composition of CD4^+^ immune cells. Thus, we employed a humanized CD4 murine knock-in model (hCD4KI) in which the extracellular fraction of the mouse CD4 antigen was replaced by the hCD4 while normal immunological function and T-cell distribution is restored (42).

### ^64^Cu-CD4-Nb1 Specifically Accumulates in CD4^+^ T Cell-Rich Organs

To generate immunoPET compatible tracers, CD4-Nb1 and GFP-Nb were labeled with the PET isotope ^64^Cu using a copper-chelating BCN-NODAGA group added to our azide-coupled Nbs. Radiolabeling yielded high radiochemical purity (≥95%) and specific binding of ^64^Cu-hCD4-Nb1 to CD4-expressing HBP-ALL cells (46.5% ± 5.6%) *in vitro* that was ~30 times higher than the non-specific binding to CD4-negative DHL cells or of the radiolabeled ^64^Cu-GFP-Nb control (**Supplementary Figure 12A**).

**Figure 6:**
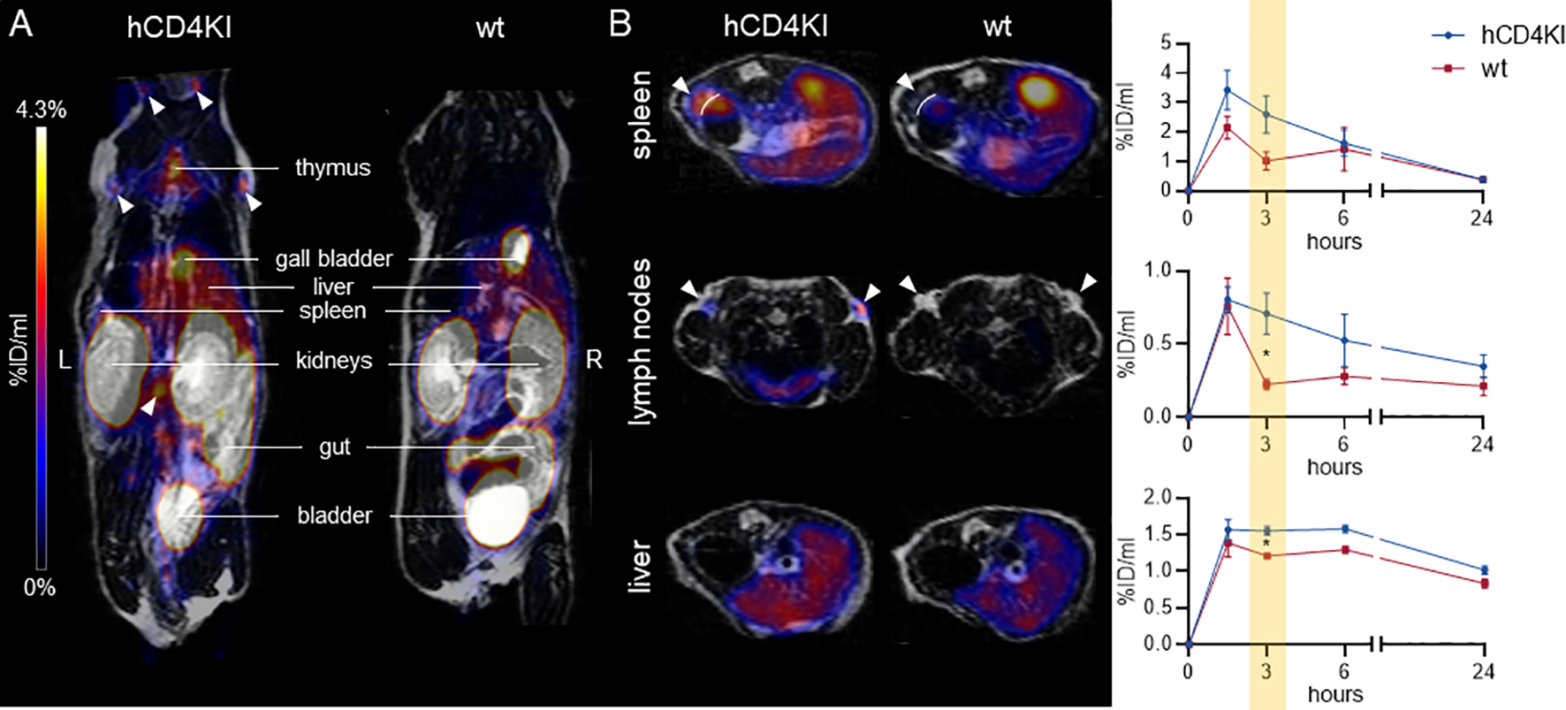
^64^Cu-CD4-Nb1 specifically accumulates in CD4^+^ T cell-rich organs. **(A)** Representative maximum intensity projection PET/MR images of human CD4 knock-in (hCD4KI) and wild-type (wt) C57BL/6 mice 3 h post intravenous (i.v.) injection of ^64^Cu-CD4-Nb1. White arrows indicate localization of lymph nodes. **(B)** Exemplary transversal PET/MR images of spleen, lymph nodes, and liver (3 h post injection) and dynamic organ uptake quantification of ^64^Cu-CD4-Nb1 over 24 h [n = 3 per group, arithmetic mean of the % injected dose per ml (%ID/ml) ± SEM, unpaired t-test of the 3-h time point, * p < 0.05]. White arrows indicate the target organ.

Subsequently, we injected ^64^Cu-CD4-Nb1 i.v. in hCD4KI and wt C57BL/6 mice and performed PET/MRI repetitively over 24 h. In two of the hCD4KI animals, we additionally followed tracer biodistribution over the first 90 min by dynamic PET (**Supplementary Figure 12B**). As expected for small-sized immunotracers, after an initial uptake peak within the first 10 min, ^64^Cu-CD4-Nb1 is rapidly cleared from the blood, lung, and liver *via* renal elimination. In comparison to wild type, mice carrying the hCD4 antigen on T cells showed an increased tracer accumulation in lymph nodes, thymus, liver, and spleen (**Figure 6A**). In these organs, which are known to harbor high numbers of CD4^+^ T cells (43), discrimination of CD4^+^-specific signal from organ background was optimal 3 h post injection (**Figure 6B**). Here, lymph nodes yielded a ~3-fold, spleen a ~2.5-fold, and liver a ~1.4-fold higher ^64^Cu-CD4-Nb1 accumulation in hCD4KI mice compared to wt littermates (**Figure 6B**). In contrast, we observed similar uptake levels for blood, muscle, lung, and kidney in both groups (**Supplementary Figure 12C**). Analyzing *ex vivo* biodistribution 24 h post tracer injection confirmed persistent accumulation of ^64^Cu-CD4-Nb1 in lymph nodes and spleen of hCD4-expressing mice, although the limited number of animals per group did not allow statistical analysis (**Supplementary Figure 12D**). In summary, these results demonstrate that CD4-Nb1 is capable of visualizing and monitoring CD4^+^ T cells in both optical and PET-based imaging.

## Discussion

Given the important role of CD4 as a marker for a variety of immune cells including monocytes, macrophages, dendritic cells, and CD4^+^ T cells, detailed monitoring of this marker is proving to be extremely important for the diagnosis and concomitant therapeutic monitoring of a variety of diseases. Several mouse studies and early clinical trials have already indicated the value of noninvasive imaging of CD4^+^ cells in rheumatoid arthritis (20), colitis (21), allogeneic stem cell transplantation (44), organ transplant rejection (45), acquired immunodeficiency disease (10), and in the context of cancer immunotherapies (46), using radiolabeled full-length antibodies or fragments thereof. However, biological activity, particularly CD4^+^ T-cell depletion, and long-term systemic retention of full-length antibodies limit their development into clinically applied immunoprobes (20, 24, 47, 48).

The aim of this study was to develop hCD4-specific Nbs as novel *in vivo* imaging probes to overcome the limitations of previous noninvasive imaging approaches. To identify binders that recognize the cellular exposed CD4, we employed two screening strategies where we selected Nbs either against adsorbed recombinant CD4 or against hCD4-expressing cells. Interestingly, both panning strategies proved successful, as demonstrated by the selection of two Nbs each that efficiently bind cell-resident CD4. Combining different biochemical analyses including epitope binning, cellular imaging, and HDX-MS, we were able to elucidate in detail the detected domains, as well as the three-dimensional epitopes addressed by the individual Nbs, and thus identified two candidates, CD4-Nb1 and CD4-Nb3, that can simultaneously bind to different segments within domain 1, while CD4-Nb2 has been shown to bind to domain 3 of CD4.

Notably, for most Nbs currently being developed for *in vivo* imaging purposes, detailed information about their epitopes is not available (49–55). There are only few examples such as the anti-HER2-specific Nb 2Rs15d, where the precise epitope was elucidated by complex crystallization (56) and that was successfully applied in a phase I study for clinical imaging (39). However, for CD4-specific Nbs, this knowledge is all the more important because epitope-specific targeting of CD4^+^ T-cell functions has far-reaching implications. This is true especially for cancer treatment, as CD4^+^ T cells have opposing effects on tumor growth and response to immunotherapies, crucially depending on the CD4 effector cell differentiation and tumor entity (57, 58). In this context, it was shown that domain 1 of CD4 mediates transient interaction of the CD4 receptor and the MHCII complex (59–61), while T-cell activation is abrogated when T-cell receptor (TCR) and CD4 colocalization is blocked *via* domain 3 (62). To further elucidate a possible impact on immunomodulation, we analyzed the effect of CD4-Nb1 and CD4-Nb4 targeting two different domains on CD4^+^ Tcell proliferation and cytokine expression. Notably, neither CD4-Nb1 nor CD4-Nb4 affected the behavior of endogenous CD4^+^ T-cells *in vitro* or induced increased cytokine levels in whole blood samples when employed at concentrations that are intended for molecular imaging purposes in patients. From these data, it can be concluded that these Nbs are mostly biologically inert and thus might be beneficial compared to full-length antibodies (24) or other antibody fragments such as the anti-CD4 Cys-diabody, which was recently reported to inhibit the proliferation of CD4^+^ cells and IFN-γ production *in vitro* (26).

Following our initial intention to generate immune tracer for *in vivo* imaging, we performed a site-directed labeling approach employing C-terminal sortagging to conjugate an azide group, which can be universally used to attach a multitude of detectable moieties by straightforward DBCO-mediated click chemistry (49). For the fluorescent and radiolabeled CD4-Nb1, we observed rapid recruitment and sustained targeting of CD4^+^ cells in a xenograft and hCD4 knock-in mouse model. Using a quantitative high-resolution PET/MR imaging approach, our radiolabeled ^64^Cu-CD4-Nb1 allowed visualizing T cell-rich organs with high sensitivity. Beyond immune organs including lymph nodes, thymus, and spleen, we could detect enhanced CD4-Nb1 uptake in liver. At this point, we cannot distinguish whether this is due to the presence of CD4^+^ cells or non-specific elimination occurring through this organ. Consequently, further experiments are needed to analyze whether CD4-Nbs lacking the Fc region are advantageous compared to larger antibody formats, which have a higher tendency to accumulate non-specifically in the spleen and liver due to Fcγ receptor-mediated uptake. However, to further modify serum retention times in order to improve specific tissue targeting, CD4-Nbs could easily be modified, as shown by the addition of an albumin-binding fragment (63) or PEGylation (49).

Considering the translation of the CD4-Nb1 for clinical imaging, additional aspects such as potential immunogenicity have to be assessed. Due to their high homology to the human type 3 VH domain, Nbs were described as weakly immunogenic in humans (64), and several strategies are available to humanize Nbs by exchanging a small number of aa residues in the framework regions (65). Moreover, a recent study of two Nbs in phase II clinical trials for PET imaging reported that very few patients developed low levels of anti-drug antibodies after prolonged administration of Nbs (66), indicating that monomeric Nbs present a low immunogenicity risk profile. In addition, long-term kidney retention of radiolabeled Nbs, mediated primarily by the endocytic receptor megalin (67), can cause undesirable nephrotoxicity and interfere with imaging of molecular targets near the kidneys. However, this can be overcome by targeted engineering of Nbs, e.g., removal of charged aa tags or simultaneous administration of positively charged components that interact with megalin receptors (68, 69). Of note, compared to other radiolabeled Nbs used for preclinical imaging of similar targets (49), CD4-Nb1 showed relatively low renal accumulation. However, as the molecular reasons for this are unclear at this stage, further studies are needed to gain deeper insight into this phenomenon.

In summary, this study demonstrates for the first time the generation and detailed characterization of Nbs specific for hCD4 and their comprehensive experimental evaluation *in vitro* and *in vivo*. In particular, CD4-Nb1 turned out as a promising candidate for a noninvasive whole-body study of CD4^+^ cells in mice. Considering the increasing importance of advanced molecular imaging in clinical practice, we anticipate that this Nb-based immunotracer could become a highly versatile tool as a novel theranostic to accompany the clinical translation of emerging immunotherapies.

## Materials and Methods

### Nanobody Library Generation

Alpaca immunization and Nb library construction were carried out as described previously (70, 71). Animal immunization has been approved by the government of Upper Bavaria (Permit number: 55.2-1-54-2532.0-80-14). In brief, an alpaca (*Vicugna pacos*) was immunized with the purified extracellular domains of hCD4 (aa26-390) recombinantly produced in HEK293 cells (antibodies-online GmbH, Germany). After initial priming with 1 mg, the animal received six boost injections with 0.5 mg hCD4 each, every second week. Then, 87 days after initial immunization, ~100 ml of blood were collected and lymphocytes were isolated by Ficoll gradient centrifugation using Lymphocyte Separation Medium (PAA Laboratories GmbH). Total RNA was extracted using TRIzol (Life Technologies), and mRNA was transcribed into cDNA using the First-Strand cDNA Synthesis Kit (GE Healthcare). The Nb repertoire was isolated in three subsequent PCR reactions using the following primer combinations: 1) CALL001 and CALL002; 2) forward primer set FR1-1, FR1-2, FR1-3, FR1-4, and reverse primer CALL002; and 3) forward primer FR1-ext1 and FR1-ext2 and reverse primer set FR4-1, FR4-2, FR4-3, FR4-4, FR4-5, and FR4-6 introducing SfiI and NotI restriction sites. The Nb library was subcloned into the SfiI/NotI sites of the pHEN4 phagemid vector (72).

### Nanobody Screening

For the selection of CD4-specific Nbs, two consecutive phage enrichment rounds were performed, both with immobilized recombinant antigen and CHO-hCD4 cells. *E. coli* TG1 cells comprising the hCD4-Nb library in pHEN4 were infected with the M13K07 helper phage to generate Nb-presenting phages. For each round, 1 × 10^11^ phages of the hCD4-Nb library were applied on immunotubes coated with hCD4 (10 µg/ml). In each selection round, extensive blocking of antigen and phages was performed with 5% milk or bovine serum albumin (BSA) in phosphate-buffered saline containing 0.05% (v/v) Tween 20 PBS-T, and with increasing panning rounds, PBS-T washing stringency was increased. Bound phages were eluted in 100 mM triethylamine (TEA; pH 10.0), followed by immediate neutralization with 1 M Tris/HCl pH 7.4. For cell-based panning, 2 × 10^6^ CHO-hCD4 or HEK293-hCD4 cells were non-enzymatically detached using dissociation buffer (Gibco) and suspended in 5% fetal bovine serum (FBS) in PBS. Antigen-expressing cells were incubated with 1 × 10^11^ phages under constant mixing at 4°C for 3 h. Cells were washed 3× with 5% FBS in PBS. Cell lines were alternated between panning rounds. Phages were eluted with 75 mM citric acid buffer at pH 2.3 for 5 min. To deplete non-CD4-specific phages, eluted phages were incubated 3× with 1 × 10^7^ wt cells. Exponentially growing *E. coli* TG1 cells were infected with eluted phages from both panning strategies and spread on selection plates for the following panning rounds. Antigen-specific enrichment for each round was monitored by counting colony-forming units (CFUs).

### Whole-Cell Phage ELISA

Polystyrene Costar 96-well cell culture plates (Corning) were coated with poly-L-lysine (Sigma Aldrich) and washed once with H_2_O. CHO-wt and CHO-hCD4 were plated at 2 × 10^4^ cells per well in 100 µl and grown to confluency overnight. Next day, 70 µl of phage supernatant was added to the culture medium of each cell type and incubated at 4°C for 3 h. Cells were washed 5× with 5% FBS in PBS. M13-horseradish peroxidase (HRP)-labeled antibody (Progen) was added at a concentration of 0.5 ng/ml for 1 h, washed 3× with 5% FBS in PBS. One-Step Ultra TMB 32048 ELISA Substrate [Thermo Fisher Scientific (TFS)] was added and incubated until color change was visible, and the reaction was stopped by addition of 100 µl of 1 M H_2_SO_4_. Detection occurred at 450 nm at a Pherastar plate reader, and phage ELISA-positive clones were defined by a 2-fold signal above wt control cells.

### Expression Constructs

The cDNA of hCD4 (UniProtKB-P01730) was amplified from hCD4-mOrange plasmid DNA (hCD4-mOrange was a gift from Sergi Padilla Parra; addgene plasmid #110192; http://n2t.net/addgene:110192; RRID : Addgene_110192) by PCR using forward primer hCD4 fwd and reverse primer hCD4 rev and introduced into BamHI and XhoI sites of a pcDNA3.1 vector variant [pcDNA3.1(+)IRES GFP, a gift from Kathleen_L Collins; addgene plasmid #51406; http://n2t.net/addgene:51406; RRID : Addgene_51406]. We replaced the neomycin resistance gene (NeoR) with the cDNA for Blasticidin S deaminase (bsd), amplified with forward primer bsd fwd and reverse primer bsd rev, by integration into the XmaI and BssHII sites of the vector. CD4 domain deletion mutants were generated using the Q5 Site-Directed Mutagenesis Kit (NEB) according to the manufacturer’s protocol. For mutants lacking domain 1 of hCD4, we introduced an N-terminal BC2-tag (35). For the generation of plasmid pcDNA3.1_CD4_ΔD1_IRES-eGFP, we used forward primer ΔD1 fwd and reverse primer ΔD1 rev; for pcDNA3.1_CD4_ΔD1ΔD2_IRES-eGFP, forward primer ΔD1ΔD2 fwd and reverse primer ΔD1ΔD2 rev; for pcDNA3.1_CD4_ΔD3ΔD4_IRES-EGFP, forward primer ΔD3ΔD4 fwd and reverse primer ΔD3ΔD4 rev. For bacterial expression of Nbs, sequences were cloned into the pHEN6 vector (73), thereby adding a C-terminal sortase tag LPETG followed by 6× His-tag for IMAC purification as described previously (34). For protein production of the extracellular domains 1-4 of hCD4 in Expi293 cells, corresponding cDNA was amplified from plasmid DNA containing full-length hCD4 cDNA (addgene plasmid #110192) using forward primer CD4-D1-4 f and reverse primer CD4-D1-4 r. A 6× His tag was introduced by the reverse primer. Esp3I and EcoRI restriction sites were used to introduce the cDNA into a pcDNA3.4 expression vector with the signal peptide MGWTLVFLFLLSVTAGVHS from the antibody JF5 (74).

### Cell Culture, Transfection, Stable Cell Line Generation

HEK293T and CHO-K1 cells were obtained from ATCC (CCL-61, LGC Standards GmbH, Germany). As this study does not include cell line-specific analysis, cells were used without additional authentication. Cells were cultivated according to standard protocols. Briefly, growth media containing Dulbecco’s modified Eagle’s medium (DMEM) (HEK293) or DMEM/F12 (CHO) [both high glucose, pyruvate (TFS)] supplemented with 10% (v/v) FBS, L-glutamine, and penicillin/streptomycin (P/S; all from TFS) were used for cultivation. Cells were passaged using 0.05% trypsin-EDTA (TFS) and were cultivated at 37°C and 5% CO_2_ atmosphere in a humidified chamber. Plasmid DNA was transfected using Lipofectamine 2000 (TFS) according to the manufacturer’s protocol. For the generation of the stable HEK293-hCD4 and CHO-hCD4 cell line, 24 h post transfection, cells were subjected to a 2-week selection period using 5 µg/ml Blasticidin S (Sigma Aldrich) followed by single cell separation. Individual clones were analyzed by live-cell fluorescence microscopy regarding their level and uniformity of GFP and CD4 expression.

### Protein Expression and Purification

CD4-specific Nbs were expressed and purified as previously published (71, 75). Extracellular fragment of hCD4 comprising domains 1–4 of hCD4 and a C-terminal His6-tag was expressed in Expi293 cells according to the manufacturer’s protocol (TFS). Cell supernatant was harvested by centrifugation 4 days after transfection, sterile filtered and purified according to previously described protocols (76). For quality control, all purified proteins were analyzed *via* sodium dodecyl sulfate-polyacrylamide gel electrophoresis (SDS-PAGE) according to standard procedures. Therefore, protein samples were denaturized (5 min, 95°C) in 2× SDS sample buffer containing 60 mM Tris/HCl, pH 6.8; 2% (w/v) SDS; 5% (v/v) 2-mercaptoethanol, 10% (v/v) glycerol, 0.02% bromphenole blue. All proteins were visualized by InstantBlue Coomassie (Expedeon) staining. For immunoblotting, proteins were transferred to a nitrocellulose membrane (Bio-Rad Laboratories) and detection was performed using anti-His primary antibody (Penta-His Antibody, #34660, Qiagen) followed by donkey anti-mouse secondary antibody labeled with Alexa Fluor 647 (Invitrogen) using a Typhoon Trio scanner (GE Healthcare, excitation 633 nm, emission filter settings 670 nm BP 30).

### Live-Cell Immunofluorescence

CHO-hCD4 and CHO wt cells transiently expressing CD4 domain-deletion mutants were plated at ~10,000 cells per well of a µClear 96-well plate (Greiner Bio One, cat. #655090) and cultivated at standard conditions. Next day, medium was replaced by live-cell visualization medium DMEMgfp-2 (Evrogen, cat. #MC102) supplemented with 10% FBS, 2 mM L-glutamine, 2 µg/ml Hoechst 33258 (Sigma Aldrich) for nuclear staining and fluorescently labeled or unlabeled CD4-Nbs at concentrations between 1 and 100 nM. Unlabeled CD4-Nbs were visualized by addition of 2.5 µg/ml anti-VHH secondary Cy5 AffiniPure Goat Anti-Alpaca IgG (Jackson ImmunoResearch). Images were acquired with a MetaXpress Micro XL system (Molecular Devices) at ×20 or ×40 magnification.

### Biolayer Interferometry

To determine the binding affinity of purified Nbs to recombinant hCD4, biolayer interferometry (BLItz, ForteBio) was performed. First, CD4 was biotinylated by 3-fold molar excess of biotin-N-hydroxysuccinimide ester. CD4 was then immobilized at single-use streptavidin biosensors (SA) according to manufacturer’s protocols. For each Nb, we executed four association/dissociation runs with concentrations appropriate for the affinities of the respective Nbs (overall between 15.6 nM and 1 µM). As a reference run, PBS was used instead of Nb in the association step. As a negative control, the GFP-Nb (500 nM) was applied in the binding studies. Recorded sensograms were analyzed using the BLItzPro software, and dissociation constants (K_D_) were calculated based on global fits. For the epitope competition analysis, two consecutive application steps were performed, with a short dissociation period of 30 s after the first association.

### Peripheral Blood Mononuclear Cell Isolation, Freezing, and Thawing

Fresh blood, buffy coats, or mononuclear blood cell concentrates were obtained from healthy volunteers at the Department of Immunology or from the ZKT Tübingen gGmbH. Participants gave informed consent, and the studies were approved by the ethical review committee of the University of Tübingen, projects 156/2012B01 and 713/2018BO2. Blood products were diluted with PBS 1× (homemade from 10× stock solution, Lonza, Switzerland), and PBMCs were isolated by density gradient centrifugation with Biocoll separation solution (Biochrom, Germany). PBMCs were washed twice with PBS 1×, counted with an NC-250 cell counter (Chemometec, Denmark), and resuspended in heat-inactivated (h.i.) FBS (Capricorn Scientific, Germany) containing 10% dimethyl sulfoxide (DMSO; Merck). Cells were immediately transferred into a -80°C freezer in a freezing container (Mr. Frosty; TFS). After at least 24 h, frozen cells were transferred into a liquid nitrogen tank and were kept frozen until use. For the experiments, cells were thawed in Iscove´s Modified Dulbecco´s Medium (IMDM) (+L-Glutamin + 25 mM HEPES; Life Technologies) supplemented with 2.5% h.i. human serum (HS; PanBiotech, Germany), 1× P/S (Sigma-Aldrich), and 50 µm β-mercaptoethanol (β-ME; Merck), washed once, counted, and used for downstream assays.

### Affinity Determination by Flow Cytometry

For cell-based affinity determination, HEK293-hCD4 cells were detached using enzyme-free cell dissociation buffer (Gibco) and resuspended in FACS buffer (PBS containing 5% FBS). For each staining condition, 200,000 cells were incubated with suitable dilution series of CD4-Nbs at 4°C for 30 min. Cells were washed two times, and for detection of Cy5 AffiniPure Goat Anti-Alpaca IgG, VHH domain (Jackson ImmunoResearch) was applied for 15 min. PBMCs (Department of Immunology/ZKT Tübingen gGmbH, Germany) were freshly thawed and resuspended in FACS buffer. For each sample, 200,000 cells were incubated with suitable concentrations of CD4-Nbs coupled to CF568 in combination with 1:500 dilution of anti-CD3-FITC (BD Biosciences) at 4°C for 30 min. For control staining, PE/Cy5-labeled anti-hCD4 antibody (RPA-T4, BioLegend) was used. After two washing steps, samples were resuspended in 200 µl FACS buffer and analyzed with a BD FACSMelody Cell Sorter. Final data analysis was performed *via* FlowJo10^®^ software (BD Biosciences).

### Sortase Labeling of Nanobodies

Sortase A pentamutant (eSrtA) in pET29 was a gift from David Liu (Addgene plasmid # 75144) and was expressed and purified as described (77). CF568-coupled peptide H-Gly-Gly-Gly-Doa-Lys-NH_2_ (sortase substrate) was custom-synthesized by Intavis AG. For the click chemistry, a peptide H-Gly-Gly-Gly-propyl-azide was synthesized. In brief, for sortase coupling 50 μM Nb, 250 μM sortase peptide dissolved in sortase buffer (50 mM Tris, pH 7.5, and 150 mM NaCl) and 10 μM sortase were mixed in coupling buffer (sortase buffer with 10 mM CaCl_2_) and incubated for 4 h at 4°C. Uncoupled Nb and sortase were depleted by IMAC. Unbound excess of unreacted sortase peptide was removed using Zeba Spin Desalting Columns (TFS, cat. #89890). Azide-coupled Nbs were labeled by strain-promoted azide-alkyne cycloaddition (SPAAC) click chemistry reaction with 2-fold molar excess of DBCO-Cy5.5 (Jena Bioscience) for 2 h at 25°C. Excess DBCO-Cy5.5 was subsequently removed by dialysis (GeBAflex-tube, 6-8 kDa, Scienova). Finally, to remove untagged Nb (side product of the sortase reaction), we used hydrophobic interaction chromatography (HIC; HiTrap Butyl-S FF, Cytiva). Binding of DBCO-Cy5.5-coupled Nb occurred in 50 mM H_2_NaPO_4_, 1.5 M (NH4)_2_SO_4_, pH7.2. Elution took place with 50 mM H_2_NaPO_4_, pH 7.2. Dye-labeled protein fractions were analyzed by SDS-PAGE followed by fluorescent scanning on a Typhoon Trio (GE-Healthcare; CF568: excitation 532 nm, emission filter settings 580 nm BP 30; Cy5.5: excitation 633 nm, emission filter settings 670 nm BP 30; 546) and subsequent Coomassie staining. Identity and purity of final products were determined by liquid chromatography-mass spectrometry (LC-MS) (CD4-Nbs-CF568, >60%; CD4-Nb1-Cy5.5, ~94%; CD4-Nb4-Cy5.5, ~99%; GBP-Cy5.5, ~94%; CD4-Nb1-3, ~99%; bivGFP-Nb, ~99%).

### Hydrogen–Deuterium Exchange

#### CD4 Deuteration Kinetics and Epitope Elucidation

On the basis of the affinity constants of 5.1 nM (CD4-Nb1), 6.5 nM (CD4-Nb2), and 75.3 nM (CD4-Nb3) (predetermined by BLI analysis), the molar ratio of antigen to Nb was calculated, ensuring 90% complex formation according to Kochert et al. (78). CD4 (5 µl, 65.5 µM) was preincubated with CD4-specific Nbs (5 µl; 60.3, 67.4, and 143.1 µM for Nb1, Nb2, and Nb3, respectively) for 10 min at 25°C. Deuteration samples containing CD4 only were preincubated with PBS instead of the Nbs. HDX of the preincubated samples was initiated by 1:10 dilution with PBS (pH 7.4) prepared with D_2_O, leading to a final concentration of 90% D_2_O. After 5- and 50-min incubation at 25°C, aliquots of 20 µl were taken and quenched by adding 20 µl ice-cold quenching solution (0.2 M TCEP with 1.5% formic acid and 4 M guanidine HCl in 100 mM ammonium formate solution pH 2.2), resulting in a final pH of 2.5. Quenched samples were immediately snap-frozen.

Immobilized pepsin (TFS) was prepared using 60 µl of 50% slurry (in ammonium formate solution pH 2.5) that was then centrifuged (1,000 × g for 3 min at 0°C). The supernatant was discarded. Prior to each analysis, samples were thawed and added to the pepsin beads. After digestion for 2 min in a water ice bath, samples were separated from the beads by centrifugation at 1,000 × g for 30 s at 0°C using a 0.22-µm filter (Merck, Millipore) and immediately analyzed by LC-MS. Undeuterated control samples for each complex and CD4 alone were prepared under the same conditions using H_2_O instead of D_2_O. Additionally, each Nb was digested without addition of CD4 to generate a list of peptic peptides deriving from the Nb. The HDX experiments of the CD4-Nb complexes were performed in triplicate. The back-exchange of the method as determined using a standard peptide mixture of 14 synthetic peptides was 24%.

### Chromatography and Mass Spectrometry

HDX samples were analyzed as described previously (75).

#### HDX Data Analysis

A peptic peptide list was generated in a preliminary LC-MS/MS experiment as described previously (75). For data-based search, no enzyme selectivity was applied; furthermore, identified peptides were manually evaluated to exclude peptides originated through cleavage after arginine, histidine, lysine, proline, and the residue after proline (79). Additionally, a separate list of peptides for each Nb was generated, and peptides overlapping in mass, retention time, and charge with the antigen digest were manually removed. Analysis of the deuterated samples was performed in MS mode only, and HDExaminer v2.5.0 (Sierra Analytics, USA) was used to calculate the deuterium uptake (centroid mass shift). HDX could be determined for peptides covering 87%–88% of the CD4 sequence (**Supplementary Figure 11**). The calculated percentages of deuterium uptake of each peptide between CD4-Nb and CD4-only were compared. Any peptide with uptake reduction of 5% or greater upon Nb binding was considered protected. All relevant HDX parameters are shown in **Supplementary Table S3** as recommended (80).

### Endotoxin Determination and Removal

The concentration of bacterial endotoxins was determined with Pierce LAL Chromogenic Endotoxin Quantitation Kit (TFS), and removal occurred using EndoTrap HD 1 ml (Lionex) according to the manufacturer’s protocols.

### Synthetic Peptides

The following human leukocyte antigen (HLA)-class II peptides were used for the stimulations: MHCII pool (HCMVA pp65 aa 109-123 MSIYVYALPLKMLNI, HCMV pp65 aa 366-382 HPTFTSQYRIQGKLEYR, EBVB9 EBNA2 aa 276-290 PRSPTVFYNIPPMPL, EBVB9 EBNA1 aa 514-527 KTSLYNLRRGTALA, EBV BXLF2 aa 126-140 LEKQLFYYIGTMLPNTRPHS, EBV BRLF1 aa 119-133 DRFFIQAPSNRVMIP, EBVB9 EBNA3 aa 381-395 PIFIRRLHRLLLMRA, EBVB9 GP350 aa 167-181 STNITAVVRAQGLDV, IABAN HEMA aa 306-318 PKYVKQNTLKLAT) or CMVpp65 aa 510-524 YQEFFWDANDIYRIF. All peptides were synthesized and dissolved in water 10% DMSO as previously described (purity ≥80%) and were kindly provided by S. Stevanović (81).

### Stimulation and Cultivation of Peripheral Blood Mononuclear Cells

PBMCs from donors previously screened for *ex vivo* CD4^+^ T-cell reactivities against MHCII peptides were thawed and rested in T-cell medium (TCM; IMDM + 1× P/S + 50 μM β-ME + 10% h.i. HS) containing 1 μg/ml DNase I (Sigma-Aldrich) at a concentration of 2–3 × 10^6^ cells/ml for 3 h at 37°C and 7.5% CO_2_. After resting, cells were washed once and counted, and up to 1 × 10^8^ cells were labeled with 1.5–2 μM carboxyfluorescein succinimidyl ester (CFSE; BioLegend, USA) in 1 ml PBS 1× for 20 min according to the manufacturer’s protocol. The cells were washed twice in medium containing 10% FBS after CFSE labeling and incubated with 5, 0.5, or 0.05 μM of CD4-Nb1, CD4-Nb4, or a control Nb for 1 h at 37°C in serum-free IMDM medium. Concentrations and duration were chosen to mimic the expected approximate concentration and serum retention time during clinical application. After incubation, cells were washed twice and counted, and each condition was separated into three parts and seeded in a 48-well cell culture plate (1.6–2.5 × 10^6^ cells/well in triplicate). Cells were stimulated with either 10 μg/ml PHA-L (Sigma-Aldrich) or 5 μg/ml MHCII peptide(s) or left unstimulated and cultured at 37°C and 7.5% CO_2_. Then, 2 ng/ml recombinant human IL-2 (R&D, USA) were added on days 3, 5, and 7. One-third of the culture on day 4, one half of the culture on days 6 and 8, and the remaining cells on day 12 were harvested, counted, and stained for flow cytometry analyses. For donor 1, the proliferation/activation status and cytokine production were analyzed in two different experiments, whereas for donors 2 and 3, cells from a single experiment were used for the three assays.

### Assessment of T-Cell Proliferation and Activation

Cells from days 4, 6, and 8 were transferred into a 96-well round-bottom plate and washed twice with FACS buffer [PBS + 0.02% sodium azide (Roth, Germany) + 2 mM EDTA (Sigma-Aldrich) + 2% h.i. FBS]. Extracellular staining was performed with CD4 APC-Cy7 (RPA-T4; BD Biosciences), CD8 BV605 (RPA-T8; BioLegend), the dead cell marker Zombie Aqua (BioLegend), CD25 PE-Cy7 (BC96; BioLegend), CD69 PE (FN50; BD Biosciences) and incubated for 20 min at 4°C. All antibodies were used at pretested optimal concentrations. Cells were washed twice with FACS buffer. Approximately 500,000 cells were acquired on the same day using an LSRFortessaTM SORP (BD Biosciences, USA) equipped with the DIVA Software (Version 6, BD Biosciences, USA). The percentage of proliferating CD4^+^ cells was determined by assessment of CFSE-negative cells and activation by the percentage of CD69^+^ or CD25^+^.

### Assessment of T-Cell Function by Intracellular Cytokine Staining

On day 12, the MHCII peptide(s)-stimulated and cultured cells were transferred into a 96-well round-bottom plate (0.5–1 × 10^6^ cells/well) and restimulated using 10 µg/ml of the same peptide(s), 10 µg/ml staphylococcal enterotoxin B (SEB; Sigma-Aldrich; positive control), or 10% DMSO (negative control). Protein transport inhibitors brefeldin A (10 µg/ml; Sigma-Aldrich) and Golgi Stop (BD Biosciences) were added at the same time as the stimuli. After 14-h stimulation at 37°C and 7.5% CO_2_, cells were stained extracellularly with the fluorescently labeled antibodies CD4 APC-Cy7, CD8 BV605, and Zombie Aqua and incubated for 20 min at 4°C. Afterward, cells were washed once, fixed, and permeabilized using the BD Cytofix/Cytoperm solution (BD Biosciences) according to the manufacturer’s instructions; stained intracellularly with TNF Pacific Blue (Mab11), IL-2 PE-Cy7 (MQ1-17H12), IFN-γ Alexa Fluor 700 (4S.B7), and CD154 APC (2431) antibodies (all BioLegend) (82); and washed twice. Approximately 500,000 cells were acquired on the same day using an LSRFortessaTM SORP (BD Biosciences, USA) equipped with the DIVA Software (Version 6; BD Biosciences). All flow cytometry analyses were performed with FlowJo version 10.6.2; gating strategies are shown in **Supplementary Figure 6**. Statistical analyses were performed with GraphPad Prism version 9.0.0.

### Full Blood Stimulation and Cytokine Release Assay

Here, 100 μl of lithium-heparin blood was incubated for 1 h at 37°C and 7.5% CO_2_. The blood was stimulated with 5 μM Nb (CD4-Nb1, CD4-Nb4, or control Nb), 100 ng/ml LPS (Invivogen, USA), or 2 μg/ml PHA-L in a final volume of 250 μl (serum-free IMDM medium) or left unstimulated for 24 h at 37°C and 7.5% CO_2_. After two centrifugations, supernatant was collected without transferring erythrocytes. The supernatants were frozen at -80°C until cytokine measurements. Levels of IL-1β, IL-1Ra, IL-4, IL-6, IL-8, IL-10, IL-12p70, IL-13, granulocyte-macrophage colony-stimulating factor (GM-CSF), IFN-γ, macrophage chemotactic protein (MCP)-1, macrophage inflammatory protein (MIP)-1β, TNFα, and vascular endothelial growth factor (VEGF) were determined using a set of in-house-developed Luminex-based sandwich immunoassays each consisting of commercially available capture and detection antibodies and calibrator proteins. All assays were thoroughly validated ahead of the study with respect to accuracy, precision, parallelism, robustness, specificity, and sensitivity (83, 84). Samples were diluted at least 1:4 or higher. After incubation of the prediluted samples or calibrator protein with the capture coated microspheres, beads were washed and incubated with biotinylated detection antibodies. Streptavidin-phycoerythrin was added after an additional washing step for visualization. For control purposes, calibrators and quality control samples were included on each microtiter plate. All measurements were performed on a Luminex FlexMap^®^ 3D analyzer system using Luminex xPONENT^®^ 4.2 software (Luminex, USA). For data analysis, MasterPlex QT, version 5.0, was employed. Standard curve and quality control samples were evaluated according to internal criteria adapted to the Westgard Rules (85) to ensure proper assay performance.

### Analysis of Cross-Species Reactivity Binding to Mouse CD4^+^ Cells by Flow Cytometry

Murine CD4^+^ cells were isolated from spleen and lymph nodes of C57BL/6N mice by positive selection over CD4 magnetic microbeads (Miltenyi Biotech, Germany). Human CD4^+^ cells were extracted from blood using StraightFrom^®^ Whole Blood CD4 MicroBeads (Miltenyi Biotech). Single-cell suspensions were incubated with 0.75 µg/ml of CD4-Nbs-Cy5.5 (~47–60 nM) or GFP-Nb-Cy5.5 (~51 nM) in 1% FPS/PBS at 4°C for 20 min and subsequently analyzed on an LSR-II cytometer (BD Biosciences).

### Optical Imaging of CD4-Expressing HPB-ALL Tumors

Human T-cell leukemia HPB-ALL cells (German Collection of Microorganisms and Cell Cultures GmbH, DSMZ, Braunschweig, Germany) were cultured in RPMI-1640 supplemented with 10% FBS and 1% P/S. Here, 10^7^ HPB-ALL cells were injected subcutaneously in the right upper flank of 7-week-old NSG (NOD.Cg-*Prkdc^scid^ Il2rg^tm1WjI^*/SzJ; Charles River Laboratories, Sulzfeld, Germany) mice, and tumor growth was monitored for 2–3 weeks. When tumors reached a diameter of ~7 mm, 5 µg of CD4-Nbs-Cy5.5 or control Nb (GFP-Nb-Cy5.5) were administered into the tail vein of two mice each. Optical imaging (OI) was performed repetitively in short-term isoflurane anesthesia over a period of 24 h using the IVIS Spectrum *In Vivo* Imaging System (PerkinElmer, Waltham, MA, USA). Four days after the first Nb administration, the CD4-Nbs-Cy5.5 groups received the GFP-Nb-Cy5.5 (and *vice versa*), and tumor biodistribution was analyzed identically by OI over 24 h. After the last imaging time point, animals were sacrificed and tumors were explanted for *ex vivo* OI analysis. Data were analyzed with Living Image 4.4 software (PerkinElmer). The fluorescence intensities were quantified by drawing regions of interest around the tumor borders and were expressed as average radiant efficiency (photons/s)/(μW/cm^2^) subtracted by the background fluorescence signal before Nb injection to eliminate potential residual signal from the previous Nb application. All mouse experiments were performed according to the German Animal Protection Law and were approved by the local authorities (Regierungspräsidium Tübingen).

### Immunofluorescence Staining of Explanted Xenograft Tumors

Freshly frozen 5-µm sections of hCD4-Nb1-Cy5.5-containing mouse tumors were analyzed using an LSM 800 laser scanning microscope (Zeiss). Afterward, the sections were fixed with perjodate-lysine-paraformaldehyde, blocked using donkey serum, and stained with primary rabbit-anti-CD4 antibody (Cell Marque, USA). Bound antibody was visualized using donkey-anti-rabbit-Cy3 secondary antibody (Dianova, Germany). YO-PRO-1 (Invitrogen, USA) was used for nuclear staining. Acquired images of the same areas were manually overlaid.

### Radiolabeling With NODAGA and ^64^Cu

All procedures for conjugation and radiolabeling with ^64^Cu were performed using metal-free equipment and Chelex 100 (Sigma-Aldrich) pretreated buffers. Azide-modified Nbs (100 µg) were treated with 4 µl of 5 mM EDTA in 250 mM sodium acetate buffer (pH 6) for 30 min at room temperature (RT). The protein was reacted with 15 molar equivalents of BCN-NODAGA (CheMatech, Dijon, France) in 250 mM sodium acetate pH 6 for 30 min at RT followed by incubation at 4°C for 18 h. Excess of chelator was removed by ultrafiltration (Amicon Ultra 0.5 ml, 3 kDa MWCO, Merck Millipore) using the same buffer. [^64^Cu]CuCl_2_ (150 MBq in 0.1 M HCl) was neutralized by addition of 1.5 volumes of 0.5 M ammonium acetate solution (pH 6), resulting in a pH of 5.5. To this solution, 50 µg of conjugate was added and incubated at 42°C for 60 min. Then, 1 µl of 20% diethylenetriaminepentaacetic acid (DTPA) solution was added to quench the labeling reaction. Complete incorporation of the radioisotope was confirmed after each radiosynthesis by thin-layer chromatography (iTLC-SA; Agilent Technologies; mobile phase 0.1 M citric acid pH 5) and high-performance size exclusion chromatography (HPSEC; BioSep SEC-s2000, 300 × 7.8 mm, Phenomenex; mobile phase DPBS with 0.5 mM EDTA). All radiolabeled preparations used for *in vivo* PET imaging had radiochemical purities of ≥97% (iTLC) and ≥94% (HPSEC).

### *In Vitro* Radioimmunoassay

Here, 10^7^ HPB-ALL or DHL cells were incubated in triplicate with 1 ng (3 MBq/µg) of radiolabeled ^64^Cu-CD4-Nb1 or ^64^Cu-GFP-Nb for 1 h at 37°C and washed twice with PBS/2% FCS. The remaining cell-bound radioactivity was measured using a Wizard² 2480 gamma counter (PerkinElmer Inc., Waltham, MA, USA) and quantified as percentage of total added activity.

### PET/MRI

Human CD4 knock-in (hCD4KI, genOway, Lyon, France) and wt C57BL/6J mice (Charles River) were injected intravenously with 5 µg (~15 MBq) of ^64^Cu-CD4-Nb1. During the scans, mice were anesthetized with 1.5% isoflurane in 100% oxygen and warmed by water-filled heating mats. Ten-minute static PET scans were performed after 1.5, 3, 6, and 24 h in a dedicated small-animal Inveon microPET scanner (Siemens Healthineers, Knoxville, TN, USA; acquisition time: 600 s). For anatomical information, sequential T2 TurboRARE MR images were acquired immediately after the PET scans on a small animal 7 T ClinScan magnetic resonance scanner (Bruker BioSpin GmbH, Rheinstetten, Germany). Dynamic PET data of the first 90 min post injection were gained in two mice and divided into 10-min frames. After attenuation correction by a cobalt-57 point source, PET images were reconstructed using an ordered subset expectation maximization (OSEM3D) algorithm and analyzed with Inveon Research Workplace (Siemens Preclinical Solutions). The volumes of interest of each organ were drawn based on the anatomical MRI to acquire corresponding PET tracer uptake. The resulting values were decay-corrected and presented as percentage of injected dose per volume (%ID/ml). *Ex vivo* γ-counting was conducted after the last imaging time point by measuring the weight and radioactivity of each organ. For quantification, standardized aliquots of the injected radiotracer were added to the measurement.

### Analyses and Statistics

Data analysis of the flow cytometry data was performed with the FlowJo Software Version 10.6.2 (FlowJo LLT, USA), and graph preparation and statistical analysis were performed using the GraphPad Prism Software (Version 8.3.0 or higher).

## Data Availability Statement

The original contributions presented in the study are included in the article/**Supplementary Material**. Further inquiries can be directed to the corresponding author.

## Ethics Statement

The animal study was reviewed and approved by Regierungspräsidium Tübingen Konrad-Adenauer-Straße 20 72072 Tübingen.

## Author Contributions

BT, MK, DSo, BP, and UR designed the study. SN and AS immunized the animal. PK, SH, and YP performed Nb selection. PK, BT, MW, TW, and AM performed biochemical characterization and functionalization of Nbs. MG and AZ performed HDX-MS analysis. AZ and MG performed HDX-MS experiments. JR, CG, MJ, and NS-M analyzed the Nb effects on T-cell proliferation and cytokine expression. DSe, AM, and SP radiolabeled the Nbs. SP and DSo performed *in vivo* imaging. MS performed staining of xenograft cryosections. BT, JR, CG, MK, BP, DSo, and UR drafted the article. MK, BP, and UR supervised the study. All authors contributed to the article and approved the submitted version.

## Funding

This work received financial support from the State Ministry of Baden-Wuerttemberg for Economic Affairs, Labour and Tourism (Grant: Predictive diagnostics of immune-associated diseases for personalized medicine. FKZ: 35-4223.10/8). We acknowlegde support by Open Access Publishing Fund of University of Tuebingen.

## Conflict of Interest

DSo, MK, BP, BT, PK, and UR are named as inventors on a patent application claiming the use of the described nanobodies for diagnosis and therapeutics filed by the Natural and Medical Sciences Institute and the Werner Siemens Imaging Center.

The remaining authors declare that the research was conducted in the absence of any commercial or financial relationships that could be construed as a potential conflict of interest.

## Publisher’s Note

All claims expressed in this article are solely those of the authors and do not necessarily represent those of their affiliated organizations, or those of the publisher, the editors and the reviewers. Any product that may be evaluated in this article, or claim that may be made by its manufacturer, is not guaranteed or endorsed by the publisher.

## References

[1] Delhalle S Bode SFN Balling R Ollert M He FQ. A Roadmap Towards Personalized Immunology. NPJ Syst Biol Appl (2018) 4:9. doi: 10.1038/s41540-017-0045-9 29423275 PMC5802799

[2] Rossi JF Ceballos P Lu ZY. Immune Precision Medicine for Cancer: A Novel Insight Based on the Efficiency of Immune Effector Cells. Cancer Commun (Lond) (2019) 39(1):34. doi: 10.1186/s40880-019-0379-3 31200766 PMC6567551

[3] Scheuenpflug J. Precision Medicine in Oncology and Immuno-Oncology: Where We Stand and Where We’re Headed. BioMed Hub (2017) 2(Suppl 1):79–86. doi: 10.1159/000481878 31988938 PMC6945891

[4] Collman R Godfrey B Cutilli J Rhodes A Hassan NF Sweet R. Macrophage-Tropic Strains of Human Immunodeficiency Virus Type 1 Utilize the CD4 Receptor. J Virol (1990) 64(9):4468–76. doi: 10.1128/JVI.64.9.4468-4476.1990 PMC247917 2200889

[5] Claeys E Vermeire K. The CD4 Receptor: An Indispensable Protein in T Cell Activation and A Promising Target for Immunosuppression. Arch Microbiol Immunol (2019) 3(3):133–50. doi: 10.26502/ami.93650036

[6] Chitnis T. The Role of CD4 T Cells in the Pathogenesis of Multiple Sclerosis. Int Rev Neurobiol (2007) 79:43–72. doi: 10.1016/S0074-7742(07)79003-7 17531837 PMC7112308

[7] Goverman J. Autoimmune T Cell Responses in the Central Nervous System. Nat Rev Immunol (2009) 9(6):393–407. doi: 10.1038/nri2550 19444307 PMC2813731

[8] Becker W Emmrich F Horneff G Burmester G Seiler F Schwarz A. Imaging Rheumatoid Arthritis Specifically With Technetium 99m CD4-Specific (T-Helper Lymphocytes) Antibodies. Eur J Nucl Med (1990) 17(3-4):156–9. doi: 10.1007/BF00811445 2149102

[9] Borst J Ahrends T Babala N Melief CJM Kastenmuller W. CD4(+) T Cell Help in Cancer Immunology and Immunotherapy. Nat Rev Immunol (2018) 18(10):635–47. doi: 10.1038/s41577-018-0044-0 30057419

[10] Di Mascio M Paik CH Carrasquillo JA Maeng JS Jang BS Shin IS. Noninvasive *In Vivo* Imaging of CD4 Cells in Simian-Human Immunodeficiency Virus (SHIV)-Infected Nonhuman Primates. Blood (2009) 114(2):328–37. doi: 10.1182/blood-2008-12-192203 PMC2714208 19417212

[11] Byrareddy SN Arthos J Cicala C Villinger F Ortiz KT Little D. Sustained Virologic Control in SIV+ Macaques After Antiretroviral and Alpha4beta7 Antibody Therapy. Science (2016) 354(6309):197–202. doi: 10.1126/science.aag1276 27738167 PMC5405455

[12] Aubert RD Kamphorst AO Sarkar S Vezys V Ha SJ Barber DL. Antigen-Specific CD4 T-Cell Help Rescues Exhausted CD8 T Cells During Chronic Viral Infection. Proc Natl Acad Sci USA (2011) 108(52):21182–7. doi: 10.1073/pnas.1118450109 PMC3248546 22160724

[13] Penaloza-MacMaster P Barber DL Wherry EJ Provine NM Teigler JE Parenteau L. Vaccine-Elicited CD4 T Cells Induce Immunopathology After Chronic LCMV Infection. Science (2015) 347(6219):278–82. doi: 10.1126/science.aaa2148 PMC4382081 25593185

[14] Mousset CM Hobo W Woestenenk R Preijers F Dolstra H van der Waart AB. Comprehensive Phenotyping of T Cells Using Flow Cytometry. Cytometry A (2019) 95(6):647–54. doi: 10.1002/cyto.a.23724 30714682

[15] Doan M Vorobjev I Rees P Filby A Wolkenhauer O Goldfeld AE. Diagnostic Potential of Imaging Flow Cytometry. Trends Biotechnol (2018) 36(7):649–52. doi: 10.1016/j.tibtech.2017.12.008 29395345

[16] Hartmann FJ Babdor J Gherardini PF Amir ED Jones K Sahaf B. Comprehensive Immune Monitoring of Clinical Trials to Advance Human Immunotherapy. Cell Rep (2019) 28(3):819–831 e4. doi: 10.1016/j.celrep.2019.06.049 31315057 PMC6656694

[17] Matos LL Trufelli DC de Matos MG da Silva Pinhal MA. Immunohistochemistry as an Important Tool in Biomarkers Detection and Clinical Practice. Biomark Insights (2010) 5:9–20. doi: 10.4137/bmi.s2185 20212918 PMC2832341

[18] Tay RE Richardson EK Toh HC. Revisiting the Role of CD4(+) T Cells in Cancer Immunotherapy-New Insights Into Old Paradigms. Cancer Gene Ther (2021) 28(1-2):5–17. doi: 10.1038/s41417-020-0183-x 32457487 PMC7886651

[19] Rubin RH Baltimore D Chen BK Wilkinson RA Fischman AJ. *In Vivo* Tissue Distribution of CD4 Lymphocytes in Mice Determined by Radioimmunoscintigraphy With an 111In-Labeled Anti-CD4 Monoclonal Antibody. Proc Natl Acad Sci USA (1996) 93(15):7460–3. doi: 10.1073/pnas.93.15.7460 PMC38766 8755495

[20] Steinhoff K Pierer M Siegert J Pigla U Laub R Hesse S. Visualizing Inflammation Activity in Rheumatoid Arthritis With Tc-99 M Anti-CD4-mAb Fragment Scintigraphy. Nucl Med Biol (2014) 41(4):350–4. doi: 10.1016/j.nucmedbio.2013.12.018 24503329

[21] Kanwar B Gao DW Hwang AB Grenert JP Williams SP Franc B. *In Vivo* Imaging of Mucosal CD4+ T Cells Using Single Photon Emission Computed Tomography in a Murine Model of Colitis. J Immunol Methods (2008) 329(1-2):21–30. doi: 10.1016/j.jim.2007.09.008 17964595 PMC2683264

[22] Dammes N Peer D. Monoclonal Antibody-Based Molecular Imaging Strategies and Theranostic Opportunities. Theranostics (2020) 10(2):938–55. doi: 10.7150/thno.37443 PMC6929980 31903161

[23] Dialynas DP Wilde DB Marrack P Pierres A Wall KA Havran W. Characterization of the Murine Antigenic Determinant, Designated L3T4a, Recognized by Monoclonal Antibody GK1.5: Expression of L3T4a by Functional T Cell Clones Appears to Correlate Primarily With Class II MHC Antigen-Reactivity. Immunol Rev (1983) 74:29–56. doi: 10.1111/j.1600-065x.1983.tb01083.x 6195085

[24] Wilde DB Marrack P Kappler J Dialynas DP Fitch FW. Evidence Implicating L3T4 in Class II MHC Antigen Reactivity; Monoclonal Antibody GK1.5 (Anti-L3T4a) Blocks Class II MHC Antigen-Specific Proliferation, Release of Lymphokines, and Binding by Cloned Murine Helper T Lymphocyte Lines. J Immunol (1983) 131(5):2178–83. 6195255

[25] Haque S Saizawa K Rojo J Janeway CA Jr. The Influence of Valence on the Functional Activities of Monoclonal Anti-L3T4 Antibodies. Discrimination of Signaling From Other Effects. J Immunol (1987) 139(10):3207–12. 2960730

[26] Freise AC Zettlitz KA Salazar FB Lu X Tavare R Wu AM. ImmunoPET Imaging of Murine CD4(+) T Cells Using Anti-CD4 Cys-Diabody: Effects of Protein Dose on T Cell Function and Imaging. Mol Imaging Biol (2017) 19(4):599–609. doi: 10.1007/s11307-016-1032-z 27966069 PMC5524218

[27] Hamers-Casterman C Atarhouch T Muyldermans S Robinson G Hamers C Songa EB. Naturally Occurring Antibodies Devoid of Light Chains. Nature (1993) 363(6428):446–8. doi: 10.1038/363446a0 8502296

[28] Lecocq Q De Vlaeminck Y Hanssens H D’Huyvetter M Raes G Goyvaerts C. Theranostics in Immuno-Oncology Using Nanobody Derivatives. Theranostics (2019) 9(25):7772–91. doi: 10.7150/thno.34941 PMC6831473 31695800

[29] Chakravarty R Goel S Cai W. Nanobody: The "Magic Bullet" for Molecular Imaging? Theranostics (2014) 4(4):386–98. doi: 10.7150/thno.8006 PMC3936291 24578722

[30] Yang EY Shah K. Nanobodies: Next Generation of Cancer Diagnostics and Therapeutics. Front Oncol (2020) 10:1182. doi: 10.3389/fonc.2020.01182 32793488 PMC7390931

[31] Chanier T Chames P. Nanobody Engineering: Toward Next Generation Immunotherapies and Immunoimaging of Cancer. Antibodies (Basel) (2019) 8(1):13. doi: 10.3390/antib8010013 PMC6640690 31544819

[32] Popp MWL Ploegh HL. Making and Breaking Peptide Bonds: Protein Engineering Using Sortase. Angew Chem Int Edition (2011) 50(22):5024–32. doi: 10.1002/anie.201008267 21538739

[33] Massa S Vikani N Betti C Ballet S Vanderhaegen S Steyaert J. Sortase A-Mediated Site-Specific Labeling of Camelid Single-Domain Antibody-Fragments: A Versatile Strategy for Multiple Molecular Imaging Modalities. Contrast Media Mol Imaging (2016) 11(5):328–39. doi: 10.1002/cmmi.1696 27147480

[34] Virant D Traenkle B Maier J Kaiser PD Bodenhofer M Schmees C. A Peptide Tag-Specific Nanobody Enables High-Quality Labeling for dSTORM Imaging. Nat Commun (2018) 9(1):930. doi: 10.1038/s41467-018-03191-2 29500346 PMC5834503

[35] Braun MB Traenkle B Koch PA Emele F Weiss F Poetz O. Peptides in Headlock–a Novel High-Affinity and Versatile Peptide-Binding Nanobody for Proteomics and Microscopy. Sci Rep (2016) 6:19211. doi: 10.1038/srep19211 26791954 PMC4726124

[36] Wu H Kwong PD Hendrickson WA. Dimeric Association and Segmental Variability in the Structure of Human CD4. Nature (1997) 387(6632):527–30. doi: 10.1038/387527a0 9168119

[37] Raybould MIJ Marks C Krawczyk K Taddese B Nowak J Lewis AP. Five Computational Developability Guidelines for Therapeutic Antibody Profiling. Proc Natl Acad Sci USA (2019) 116(10):4025–30. doi: 10.1073/pnas.1810576116 PMC6410772 30765520

[38] Xu Y Wang D Mason B Rossomando T Li N Liu D. Structure, Heterogeneity and Developability Assessment of Therapeutic Antibodies. MAbs (2019) 11(2):239–64. doi: 10.1080/19420862.2018.1553476 PMC6380400 30543482

[39] Keyaerts M Xavier C Heemskerk J Devoogdt N Everaert H Ackaert C. Phase I Study of 68Ga-HER2-Nanobody for PET/CT Assessment of HER2 Expression in Breast Carcinoma. J Nucl Med (2016) 57(1):27–33. doi: 10.2967/jnumed.115.162024 26449837

[40] Xavier C Vaneycken I D’Huyvetter M Heemskerk J Keyaerts M Vincke C. Synthesis, Preclinical Validation, Dosimetry, and Toxicity of 68Ga-NOTA-Anti-HER2 Nanobodies for iPET Imaging of HER2 Receptor Expression in Cancer. J Nucl Med (2013) 54(5):776–84. doi: 10.2967/jnumed.112.111021 23487015

[41] Masuda S Kumano K Suzuki T Tomita T Iwatsubo T Natsugari H. Dual Antitumor Mechanisms of Notch Signaling Inhibitor in a T-Cell Acute Lymphoblastic Leukemia Xenograft Model. Cancer Sci (2009) 100(12):2444–50. doi: 10.1111/j.1349-7006.2009.01328.x PMC11159026 19775286

[42] Killeen N Sawada S Littman DR. Regulated Expression of Human CD4 Rescues Helper T Cell Development in Mice Lacking Expression of Endogenous CD4. EMBO J (1993) 12(4):1547–53. doi: 10.1002/j.1460-2075.1993.tb05798.x PMC413367 8467804

[43] Sckisel GD Mirsoian A Minnar CM Crittenden M Curti B Chen JQ. Differential Phenotypes of Memory CD4 and CD8 T Cells in the Spleen and Peripheral Tissues Following Immunostimulatory Therapy. J Immunother Cancer (2017) 5:33. doi: 10.1186/s40425-017-0235-4 28428882 PMC5394626

[44] Tavare R McCracken MN Zettlitz KA Salazar FB Olafsen T Witte ON. Immuno-PET of Murine T Cell Reconstitution Postadoptive Stem Cell Transplantation Using Anti-CD4 and Anti-CD8 Cys-Diabodies. J Nucl Med (2015) 56(8):1258–64. doi: 10.2967/jnumed.114.153338 PMC4859343 25952734

[45] Li H Chen Y Jin Q Wu Y Deng C Gai Y. Noninvasive Radionuclide Molecular Imaging of the CD4-Positive T Lymphocytes in Acute Cardiac Rejection. Mol Pharm (2021) 18(3):1317–26. doi: 10.1021/acs.molpharmaceut.0c01155 33506680

[46] Kristensen LK Frohlich C Christensen C Melander MC Poulsen TT Galler GR. CD4(+) and CD8a(+) PET Imaging Predicts Response to Novel PD-1 Checkpoint Inhibitor: Studies of Sym021 in Syngeneic Mouse Cancer Models. Theranostics (2019) 9(26):8221–38. doi: 10.7150/thno.37513 PMC6857046 31754392

[47] Choy EH Panayi GS Emery P Madden S Breedveld FC Kraan MC. Repeat-Cycle Study of High-Dose Intravenous 4162W94 Anti-CD4 Humanized Monoclonal Antibody in Rheumatoid Arthritis. A Randomized Placebo-Controlled Trial. Rheumatol (Oxford) (2002) 41(10):1142–8. doi: 10.1093/rheumatology/41.10.1142 12364634

[48] Moreland LW Pratt PW Mayes MD Postlethwaite A Weisman MH Schnitzer T. Double-Blind, Placebo-Controlled Multicenter Trial Using Chimeric Monoclonal Anti-CD4 Antibody, cM-T412, in Rheumatoid Arthritis Patients Receiving Concomitant Methotrexate. Arthritis Rheum (1995) 38(11):1581–8. doi: 10.1002/art.1780381109 7488278

[49] Rashidian M Ingram JR Dougan M Dongre A Whang KA LeGall C. Predicting the Response to CTLA-4 Blockade by Longitudinal Noninvasive Monitoring of CD8 T Cells. J Exp Med (2017) 214(8):2243–55. doi: 10.1084/jem.20161950 PMC5551571 28666979

[50] Huang L Gainkam LO Caveliers V Vanhove C Keyaerts M De Baetselier P. SPECT Imaging With 99mtc-Labeled EGFR-Specific Nanobody for *In Vivo* Monitoring of EGFR Expression. Mol Imaging Biol (2008) 10(3):167–75. doi: 10.1007/s11307-008-0133-8 18297364

[51] Roovers RC Laeremans T Huang L De Taeye S Verkleij AJ Revets H. Efficient Inhibition of EGFR Signaling and of Tumour Growth by Antagonistic Anti-EFGR Nanobodies. Cancer Immunol Immunother (2007) 56(3):303–17. doi: 10.1007/s00262-006-0180-4 PMC11030579 16738850

[52] Evazalipour M D’Huyvetter M Tehrani BS Abolhassani M Omidfar K Abdoli S. Generation and Characterization of Nanobodies Targeting PSMA for Molecular Imaging of Prostate Cancer. Contrast Media Mol Imaging (2014) 9(3):211–20. doi: 10.1002/cmmi.1558 24700748

[53] Blykers A Schoonooghe S Xavier C D’Hoe K Laoui D D’Huyvetter M. PET Imaging of Macrophage Mannose Receptor-Expressing Macrophages in Tumor Stroma Using 18f-Radiolabeled Camelid Single-Domain Antibody Fragments. J Nucl Med (2015) 56(8):1265–71. doi: 10.2967/jnumed.115.156828 26069306

[54] Bala G Baudhuin H Remory I Gillis K Debie P Krasniqi A. Evaluation of [(99m)Tc]Radiolabeled Macrophage Mannose Receptor-Specific Nanobodies for Targeting of Atherosclerotic Lesions in Mice. Mol Imaging Biol (2018) 20(2):260–7. doi: 10.1007/s11307-017-1117-3 28875290

[55] Jailkhani N Ingram JR Rashidian M Rickelt S Tian C Mak H. Noninvasive Imaging of Tumor Progression, Metastasis, and Fibrosis Using a Nanobody Targeting the Extracellular Matrix. Proc Natl Acad Sci USA (2019) 116(28):14181–90. doi: 10.1073/pnas.1817442116 PMC6628802 31068469

[56] D’Huyvetter M De Vos J Xavier C Pruszynski M Sterckx YGJ Massa S. (131)I-Labeled Anti-HER2 Camelid sdAb as a Theranostic Tool in Cancer Treatment. Clin Cancer Res (2017) 23(21):6616–28. doi: 10.1158/1078-0432.CCR-17-0310 PMC5668161 28751451

[57] Bruni D Angell HK Galon J. The Immune Contexture and Immunoscore in Cancer Prognosis and Therapeutic Efficacy. Nat Rev Cancer (2020) 20(11):662–80. doi: 10.1038/s41568-020-0285-7 32753728

[58] Accogli T Bruchard M Vegran F. Modulation of CD4 T Cell Response According to Tumor Cytokine Microenvironment. Cancers (Basel) (2021) 13(3):373. doi: 10.3390/cancers13030373 PMC7864169 33498483

[59] Sakihama T Smolyar A Reinherz EL. Oligomerization of CD4 Is Required for Stable Binding to Class II Major Histocompatibility Complex Proteins But Not for Interaction With Human Immunodeficiency Virus Gp120. Proc Natl Acad Sci USA (1995) 92(14):6444–8. doi: 10.1073/pnas.92.14.6444 PMC41534 7604010

[60] Jonsson P Southcombe JH Santos AM Huo J Fernandes RA McColl J. Remarkably Low Affinity of CD4/peptide-Major Histocompatibility Complex Class II Protein Interactions. Proc Natl Acad Sci USA (2016) 113(20):5682–7. doi: 10.1073/pnas.1513918113 PMC4878507 27114505

[61] Cruikshank WW Greenstein JL Theodore AC Center DM. Lymphocyte Chemoattractant Factor Induces CD4-Dependent Intracytoplasmic Signaling in Lymphocytes. J Immunol (1991) 146(9):2928–34. 1673145

[62] Vignali DA Vignali KM. Profound Enhancement of T Cell Activation Mediated by the Interaction Between the TCR and the D3 Domain of CD4. J Immunol (1999) 162(3):1431–9. 9973399

[63] Tijink BM Laeremans T Budde M Stigter-van Walsum M Dreier T de Haard HJ. Improved Tumor Targeting of Anti-Epidermal Growth Factor Receptor Nanobodies Through Albumin Binding: Taking Advantage of Modular Nanobody Technology. Mol Cancer Ther (2008) 7(8):2288–97. doi: 10.1158/1535-7163.MCT-07-2384 18723476

[64] Muyldermans S Baral TN Retamozzo VC De Baetselier P De Genst E Kinne J. Camelid Immunoglobulins and Nanobody Technology. Vet Immunol Immunopathol (2009) 128(1-3):178–83. doi: 10.1016/j.vetimm.2008.10.299 19026455

[65] Vincke C Loris R Saerens D Martinez-Rodriguez S Muyldermans S Conrath K. General Strategy to Humanize a Camelid Single-Domain Antibody and Identification of a Universal Humanized Nanobody Scaffold. J Biol Chem (2009) 284(5):3273–84. doi: 10.1074/jbc.M806889200 19010777

[66] Ackaert C Smiejkowska N Xavier C Sterckx YGJ Denies S Stijlemans B. Immunogenicity Risk Profile of Nanobodies. Front Immunol (2021) 12:632687. doi: 10.3389/fimmu.2021.632687 33767701 PMC7985456

[67] Gainkam LO Caveliers V Devoogdt N Vanhove C Xavier C Boerman O. Localization, Mechanism and Reduction of Renal Retention of Technetium-99m Labeled Epidermal Growth Factor Receptor-Specific Nanobody in Mice. Contrast Media Mol Imaging (2011) 6(2):85–92. doi: 10.1002/cmmi.408 20936711

[68] D’Huyvetter M Vincke C Xavier C Aerts A Impens N Baatout S. Targeted Radionuclide Therapy With A 177Lu-Labeled Anti-HER2 Nanobody. Theranostics (2014) 4(7):708–20. doi: 10.7150/thno.8156 PMC4038753 24883121

[69] de Jong M Barone R Krenning E Bernard B Melis M Visser T. Megalin Is Essential for Renal Proximal Tubule Reabsorption of (111)In-DTPA-Octreotide. J Nucl Med (2005) 46(10):1696–700. 16204720

[70] Traenkle B Emele F Anton R Poetz O Haeussler RS Maier J. Monitoring Interactions and Dynamics of Endogenous Beta-Catenin With Intracellular Nanobodies in Living Cells. Mol Cell Proteomics (2015) 14(3):707–23. doi: 10.1074/mcp.M114.044016 PMC4349989 25595278

[71] Maier J Traenkle B Rothbauer U. Real-Time Analysis of Epithelial-Mesenchymal Transition Using Fluorescent Single-Domain Antibodies. Sci Rep (2015) 5:13402. doi: 10.1038/srep13402 26292717 PMC4544033

[72] Arbabi Ghahroudi M Desmyter A Wyns L Hamers R Muyldermans S. Selection and Identification of Single Domain Antibody Fragments From Camel Heavy-Chain Antibodies. FEBS Lett (1997) 414(3):521–6. doi: 10.1016/S0014-5793(97)01062-4 9323027

[73] Rothbauer U Zolghadr K Muyldermans S Schepers A Cardoso MC Leonhardt H. A Versatile Nanotrap for Biochemical and Functional Studies With Fluorescent Fusion Proteins. Mol Cell Proteomics (2008) 7(2):282–9. doi: 10.1074/mcp.M700342-MCP200 17951627

[74] Davies G Rolle AM Maurer A Spycher PR Schillinger C Solouk-Saran D. Towards Translational ImmunoPET/MR Imaging of Invasive Pulmonary Aspergillosis: The Humanised Monoclonal Antibody JF5 Detects Aspergillus Lung Infections *In Vivo* . Theranostics (2017) 7(14):3398–414. doi: 10.7150/thno.20919 PMC5596432 28912884

[75] Wagner TR Ostertag E Kaiser PD Gramlich M Ruetalo N Junker D. NeutrobodyPlex-Monitoring SARS-CoV-2 Neutralizing Immune Responses Using Nanobodies. EMBO Rep (2021) 22(5):e52325. doi: 10.15252/embr.202052325 33904225 PMC8097376

[76] Becker M Strengert M Junker D Kaiser PD Kerrinnes T Traenkle B. Exploring Beyond Clinical Routine SARS-CoV-2 Serology Using MultiCoV-Ab to Evaluate Endemic Coronavirus Cross-Reactivity. Nat Commun (2021) 12(1):1152. doi: 10.1038/s41467-021-20973-3 33608538 PMC7896075

[77] Chen I Dorr BM Liu DR. A General Strategy for the Evolution of Bond-Forming Enzymes Using Yeast Display. Proc Natl Acad Sci USA (2011) 108(28):11399–404. doi: 10.1073/pnas.1101046108 PMC3136257 21697512

[78] Kochert BA Iacob RE Wales TE Makriyannis A Engen JR. Hydrogen-Deuterium Exchange Mass Spectrometry to Study Protein Complexes. Methods Mol Biol (2018) 1764:153–71. doi: 10.1007/978-1-4939-7759-8_10 29605914

[79] Hamuro Y Coales SJ. Optimization of Feasibility Stage for Hydrogen/Deuterium Exchange Mass Spectrometry. J Am Soc Mass Spectrom (2018) 29(3):623–9. doi: 10.1007/s13361-017-1860-3 29299838

[80] Masson GR Burke JE Ahn NG Anand GS Borchers C Brier S. Recommendations for Performing, Interpreting and Reporting Hydrogen Deuterium Exchange Mass Spectrometry (HDX-MS) Experiments. Nat Methods (2019) 16(7):595–602. doi: 10.1038/s41592-019-0459-y 31249422 PMC6614034

[81] Loffler MW Nussbaum B Jager G Jurmeister PS Budczies J Pereira PL. A Non-Interventional Clinical Trial Assessing Immune Responses After Radiofrequency Ablation of Liver Metastases From Colorectal Cancer. Front Immunol (2019) 10:2526. doi: 10.3389/fimmu.2019.02526 31803175 PMC6877671

[82] Widenmeyer M Griesemann H Stevanovic S Feyerabend S Klein R Attig S. Promiscuous Survivin Peptide Induces Robust CD4+ T-Cell Responses in the Majority of Vaccinated Cancer Patients. Int J Cancer (2012) 131(1):140–9. doi: 10.1002/ijc.26365 21858810

[83] EMEA. Guideline on Bioanalytical Method Validation. European Medicines Agency; Comittee for Medicinal Products for Human Use. London (CHMP (2013).

[84] FDA. Bioanalytical Method Validation: Guidance for Industry. Silver Spring, Rockville: U.S. Department of Health and Human Services, Food and Drug Administration, Center for Drug Evaluation and Research, Center for Veterinary Medicine.

[85] Westgard JO Barry PL Hunt MR Groth T. A Multi-Rule Shewhart Chart for Quality Control in Clinical Chemistry. Clin Chem (1981) 27(3):493–501. doi: 10.1093/clinchem/27.3.493 7471403

