# Supplementary material for: Single-Domain Antibodies for Targeting, Detection, and In Vivo Imaging of Human CD4+ Cells
Source: Front Immunol. 2021 Dec 9;12:799910. doi: 10.3389/fimmu.2021.799910 (PMC8696186; doi:10.3389/fimmu.2021.799910)
Supplement: Supplementary file 1 [file DataSheet_1.pdf]

Single-domain antibodies for targeting, detection and *in vivo* imaging of human CD4$^+$ cells

Bjoern Traenkle[1], Philipp D. Kaiser[1], Stefania Pezzana[2], Jennifer Richardson[3], Marius Gramlich[1], Teresa R. Wagner[1,4], Dominik Seyfried[2,7], Melissa Weldle[4], Stefanie Holz[4], Yana Parfyonova[4], Stefan Nueske[5], Armin M. Scholz[5], Anne Zeck[1], Meike Jakobi[1], Nicole Schneiderhan-Marra[1], Martin Schaller[6], Andreas Maurer[2,8], Cécile Gouttefangeas[3,7,8], Manfred Kneilling[2,6,8], Bernd J. Pichler[2,7,8], Dominik Sonanini[2,9], and Ulrich Rothbauer[1,4,8#]

Affiliations/Addresses

[1] NMI Natural and Medical Sciences Institute at the University of Tübingen, Reutlingen, Germany

[2] Werner Siemens Imaging Center, Department of Preclinical Imaging and Radiopharmacy, University of Tübingen, Tübingen, Germany

[3] Department of Immunology, Institute of Cell Biology, University of Tübingen, Tübingen, Germany

[4] Pharmaceutical Biotechnology, Eberhard Karls University Tübingen, Germany

[5] Livestock Center of the Faculty of Veterinary Medicine, Ludwig Maximilians University Munich, Oberschleissheim, Germany

[6] Department of Dermatology, University of Tübingen, Tübingen, Germany

[7] German Cancer Consortium (DKTK) and German Cancer Research Center (DKFZ) partner site Tübingen, Tübingen, Germany

[8] Cluster of Excellence iFIT (EXC2180) "Image-Guided and Functionally Instructed Tumor Therapies", University of Tübingen, Germany[9] Department of Medical Oncology and Pneumology, University of Tübingen, Tübingen, Germany

[#] corresponding author

Prof. Dr. Ulrich Rothbauer, Natural and Medical Sciences Institute at the University of Tübingen Markwiesenstr. 55, 72770 Reutlingen, Germany.

E-mail: ulrich.rothbauer@uni-tuebingen.de

Phone: +49 7121 51530-415

Fax: +49 7121 51530-816

Orcid ID: 0000-0001-5923-8986

Supplementary Information

Supplementary Figure 1

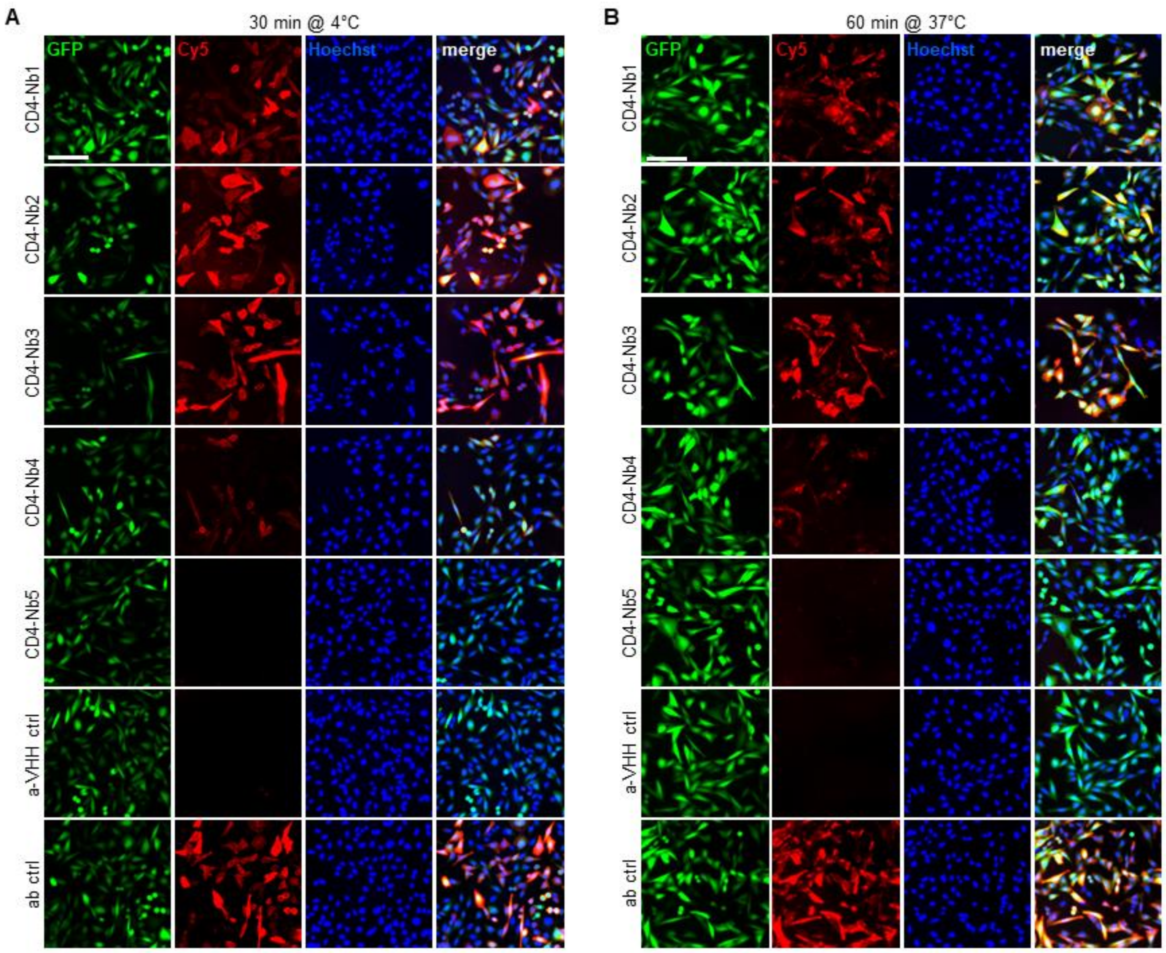

Supplementary Figure 1 Live-cell immunofluorescence staining of CHO-hCD4 cells incubated with CD4-Nbs (100 nM) followed by detection using a secondary Cy5-labeled anti-VHH antibody and 2 µg/ml Hoechst33258 for 30 min at 4°C (**A**) or 60 min at 37°C (**B**). Shown are representative images of CHO-hCD4 cells simultaneously expressing cytosolic GFP (left column) and hCD4 (second column from left) from a bicistronic mRNA. Nuclear staining and merge of channels is shown in column 3 and 4 from the left. Negative control staining using secondary Cy5-labeled anti-VHH antibody alone (a-VHH ctrl) and positive control using Cy5-labeled anti-hCD4 antibody (ab ctrl) are shown in bottom two rows. Scale bar 100 µm.

Supplementary Figure 2

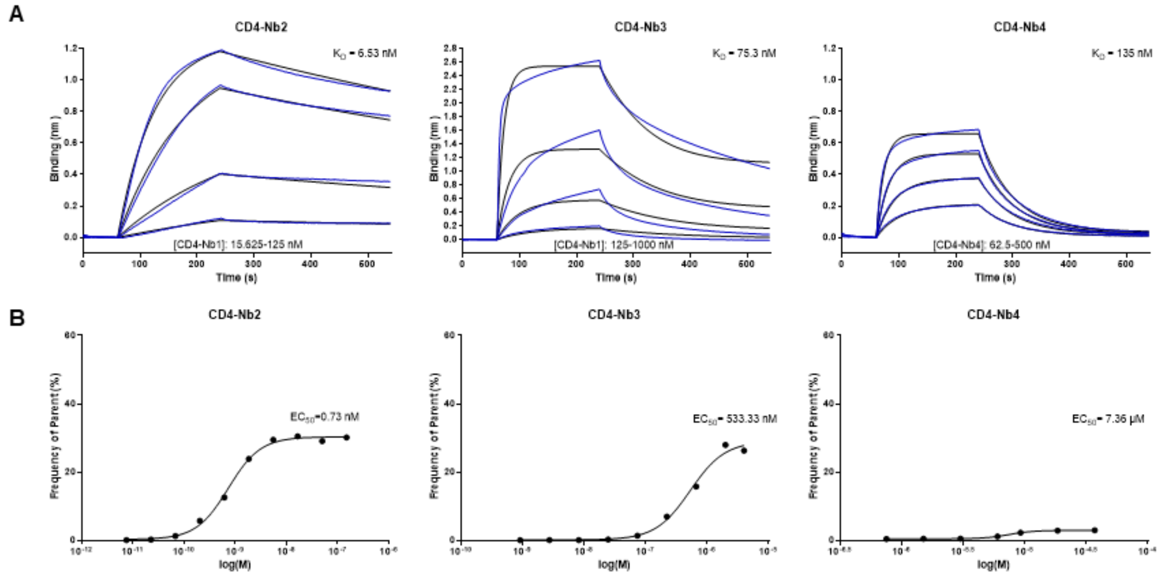

Supplementary Figure 2 Affinities of identified CD4-Nbs (**A**) Sensograms of biolayer interferometry-based affinity measurements of CD4-Nb2, CD4-Nb3 and CD4-Nb4 are shown. Biotinylated hCD4 was immobilized on streptavidin biosensors and kinetic measurements were performed by using four concentrations of purified Nbs ranging from 15.6 nM - 1 µM. (**B**) EC_{50} determination of CD4-Nbs for cellular expressed hCD4 by flow cytometry. The percentage of positively stained HEK293-hCD4 (frequency of parent) was plotted against indicated concentrations of CD4-Nbs. EC_{50} values were calculated from a four-parametric sigmoidal model.

Supplementary Figure 3

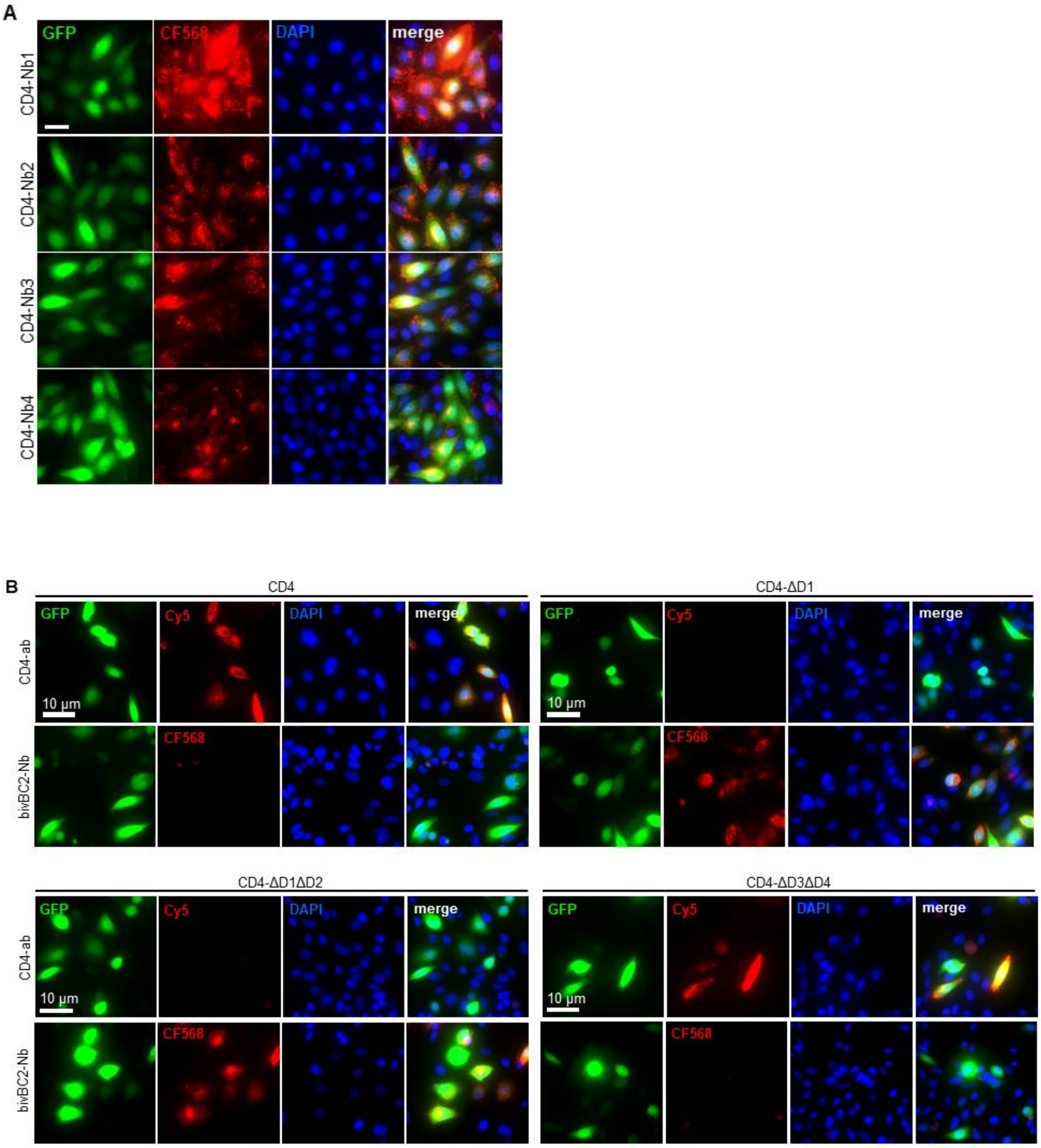

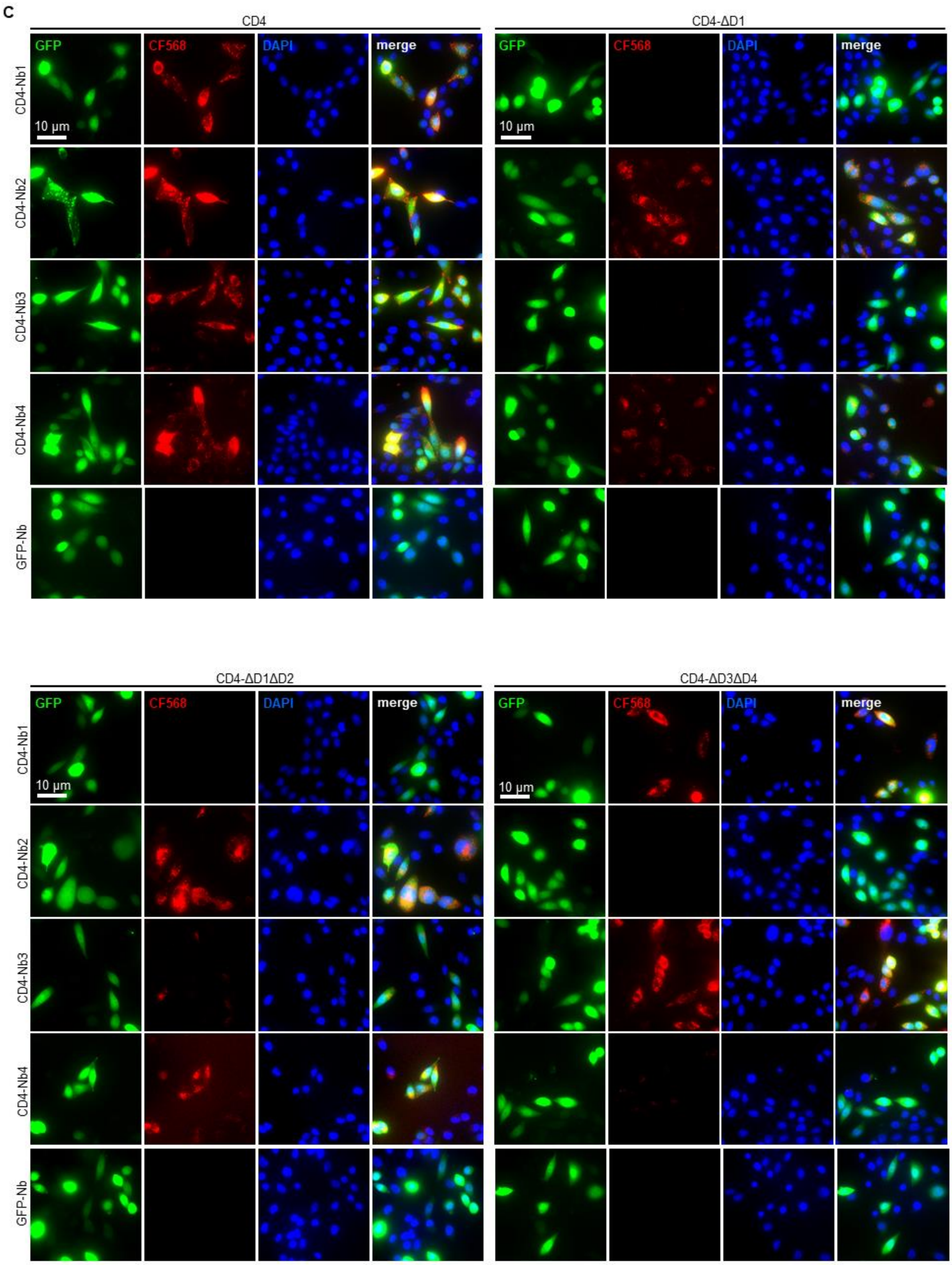

Supplementary Figure 3 CD4-Nbs bind different domains of human CD4. (**A**) Live-cell immunofluorescence staining of CHO-hCD4 cells with CD4-Nbs coupled to the fluorescent dye CF568, and 2 µg/ml Hoechst33258 for 60 min at 37°C. (**B**) Control staining of full-length hCD4 (CD4) or hCD4 domain-deletion mutants CD4-ΔD1, CD4-ΔD1ΔD2 or CD4-ΔD3ΔD4 with

fluorescently labeled anti-CD4 antibody RPA-T4-PE/Cy5 (CD4-ab) or bivalent BC2-Nb coupled to CF568 (bivBC2-Nb). (**C**) Live-cell immunofluorescence staining of CHO cells transiently expressing full-length hCD4 or hCD4 indicated domain-deletion mutants with CF568-labeled CD4-Nbs or a non-specific GFP-Nb (100 nM). Scale bars 10 µm.

Supplementary Figure 4

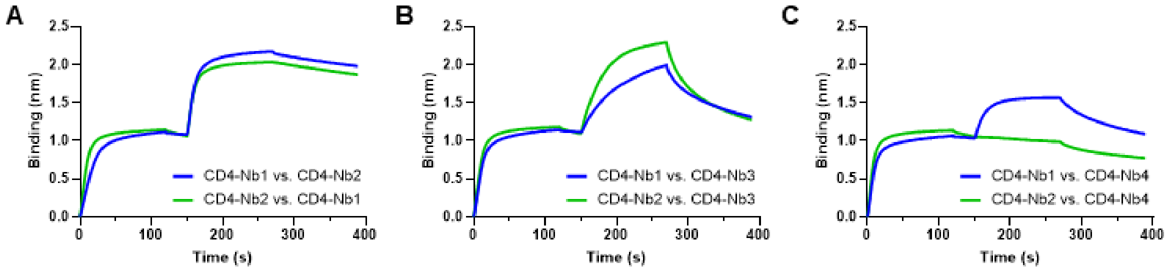

Supplementary Figure 4 Epitope binning analysis of CD4-Nbs by biolayer interferometry (BLI) (**A**) Representative BLI sensograms of single measurements of combinatorial Nb binding to the recombinant extracellular portion of hCD4 of CD4-Nb1 (blue) and CD4-Nb2 (green) with (**A**) one another, (**B**) CD4-Nb3, or (**C**) CD4-Nb4.

Supplementary Figure 5

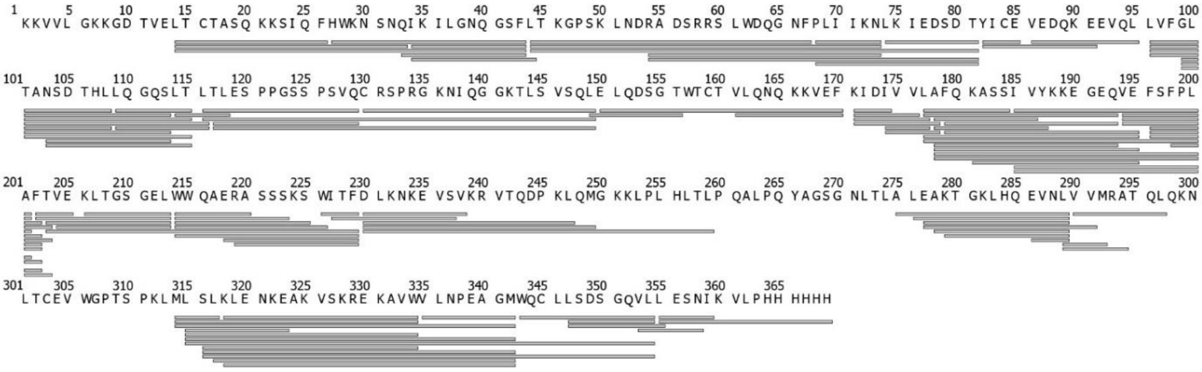

Supplementary Figure 5 Peptide sequence coverage of human CD4 for HDX-MS analysis. 116 possible peptides could be identified by MSMS (depicted as bars) leading to a sequence coverage of 88% for the HDX analysis.

Supplementary Figure 6

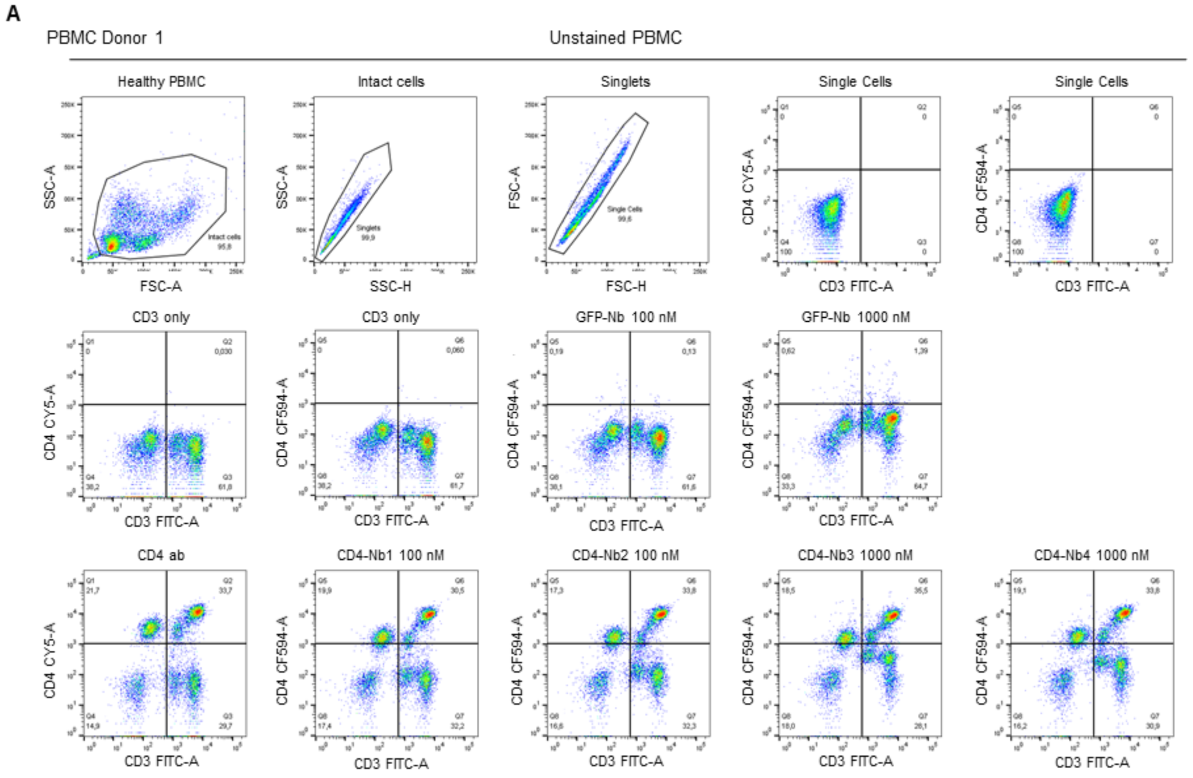

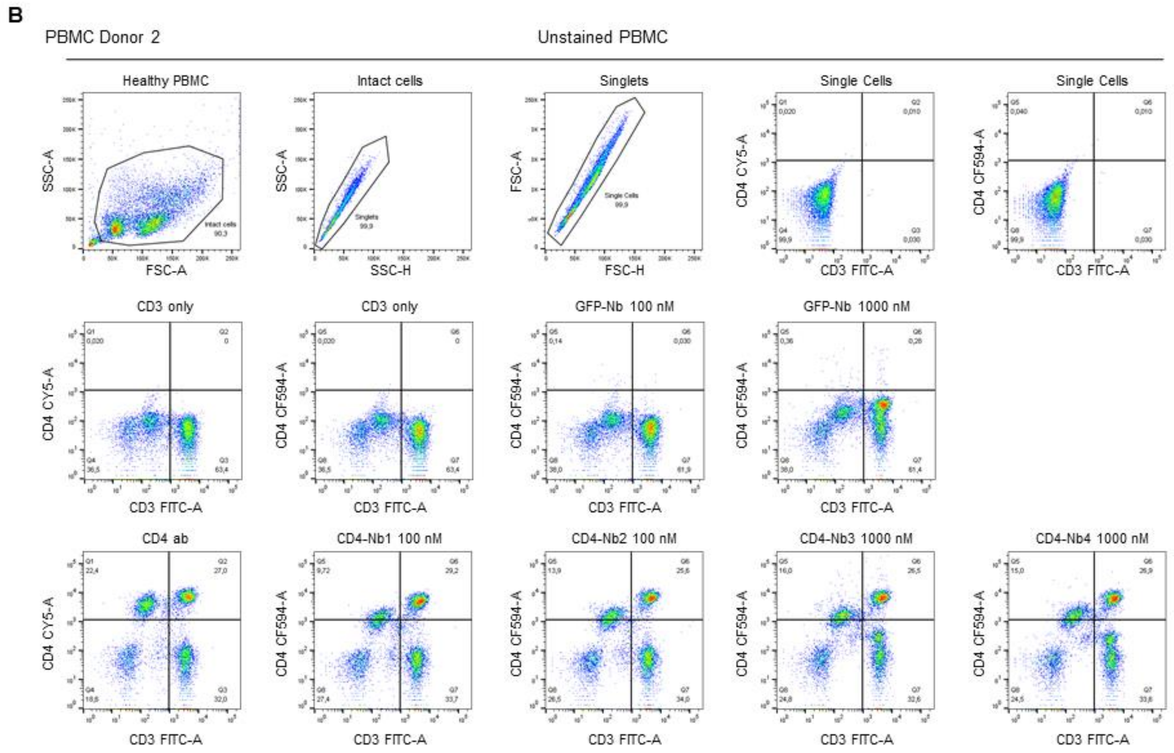

C

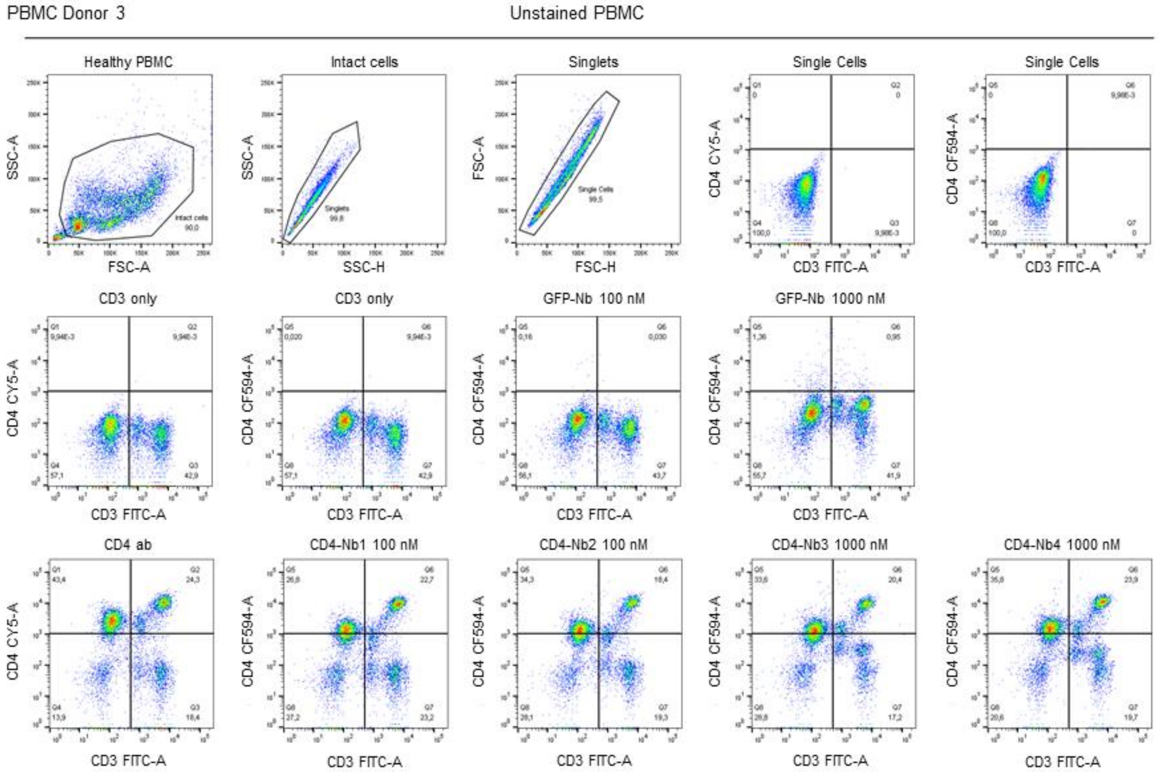

Supplementary Figure 6 Binding of CD4-Nbs to $CD4^+$ cells present in human PBMCs. Top row shows gating strategy for flow cytometry analysis of $CD4^+CD3^+$ double-positive human PBMCs. Middle and bottom row shows final gating step and quantification of these cells for donor 1 (**A**), donor 2 (**B**), and donor 3 (**C**) stained with an anti-CD4 antibody (CD4 ab), anti-GFP control Nb (GFP-Nb), or CD4-Nb1 - CD4-Nb4 at indicated concentrations.

Supplementary Figure 7

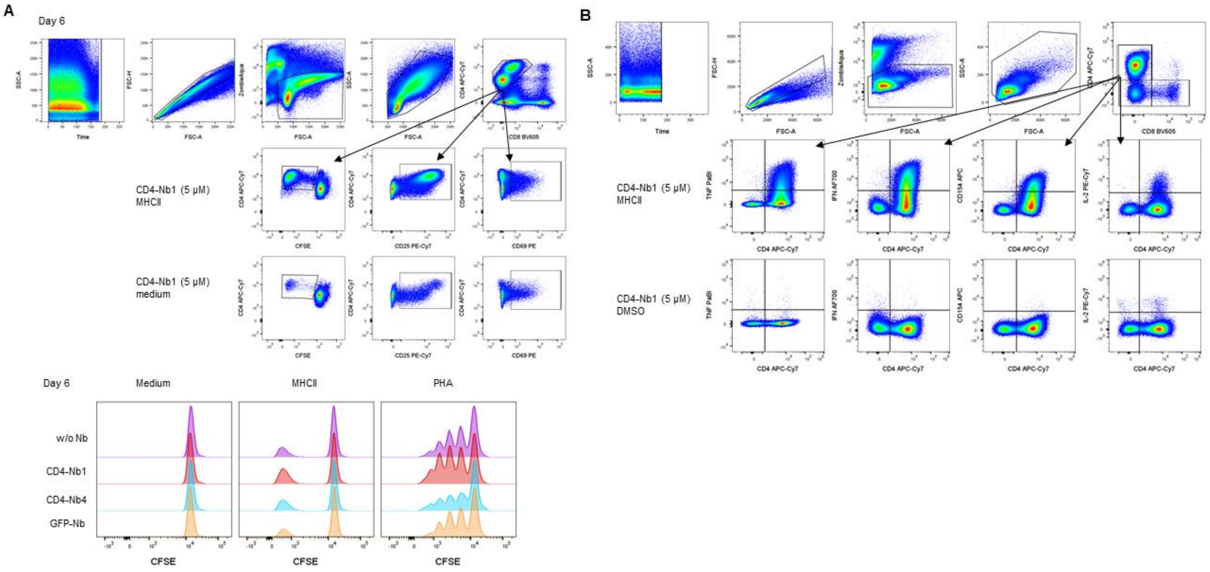

Supplementary Figure 7 Determination of the effect of CD4-Nbs on CD4$^+$ T cells. (**A**) Gating strategy for analysis of proliferation and activation of CD4$^+$ cells after stimulation. Top row from left to right: Time gate, single cells, live cells, lymphocytes, CD4$^+$ cells. Middle and bottom rows: gates were place on proliferating CFSE-low/negative CD4$^+$ cells (left), CD25$^+$CD4$^+$ cells (middle) and CD69$^+$CD4$^+$ cells (right). Histogram overlay shows the number of divisions as CFSE labeling within CD4$^+$ cells. Shown is one representative example (donor 2) on day 6. (**B**) Gating strategy (donor 3) for analysis of activation marker and cytokine expression of CD4$^+$ cells in intracellular staining after 12 days of culture and 14 h restimulation. Top row from left to right: Time gate, single cells, live cells, lymphocytes, CD4$^+$ cells, CD4/CD8 staining (gating on CD8^{neg} cells). Middle and bottom rows show the expression of TNF, IFN-γ, CD154 and IL-2 after restimulation with MHC-class II peptides or control DMSO/water.

Supplementary Figure 8

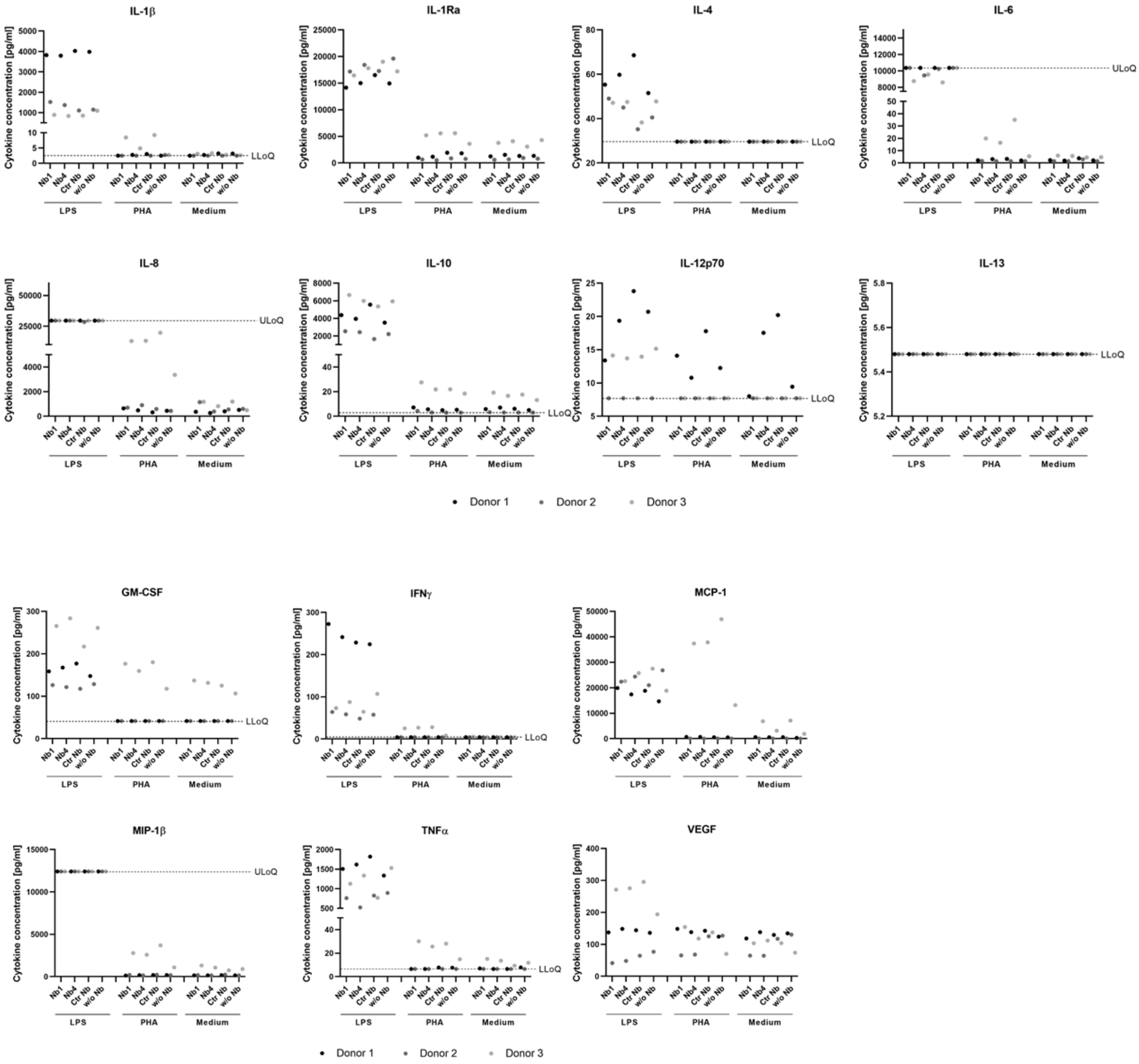

Supplementary Figure 8 Determination of cytokines secreted from whole blood samples of donors after treatment with CD4-Nbs. Blood samples of three donors were incubated with 5 µM CD4-Nb1, CD4-Nb4, GFP-Nb (control) or w/o Nb and stimulated with lipopolysaccharide (LPS), phytohaemagglutinin (PHA) or medium only as control. Secreted cytokines (listed in table S2) were measured and quantified using an in-house developed microsphere-based (Luminex) multiplex sandwich immunoassay. Results of one biological experiment are shown as colored dots indicating measured cytokine levels of one individual.

Supplementary Figure 9

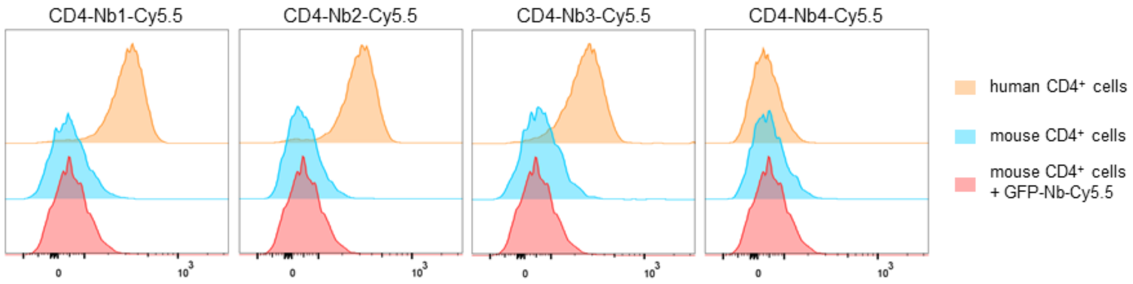

Supplementary Figure 9 Cross-species reactivity testing of Cy5.5-labeled CD4-Nbs. Flow cytometry of human and mouse $CD4^{+}$ cells stained with CD4-Nbs-Cy5.5 or GFP-Nb-Cy5.5. Binding to human CD4 was confirmed for CD4-Nb1, CD4-Nb2 and CD4-Nb3. CD4-Nb4 did not show staining at this concentration (0.75 µg/ml, ~49 nM). None of the tested CD4-Nbs stained murine CD4.

Supplementary Figure 10

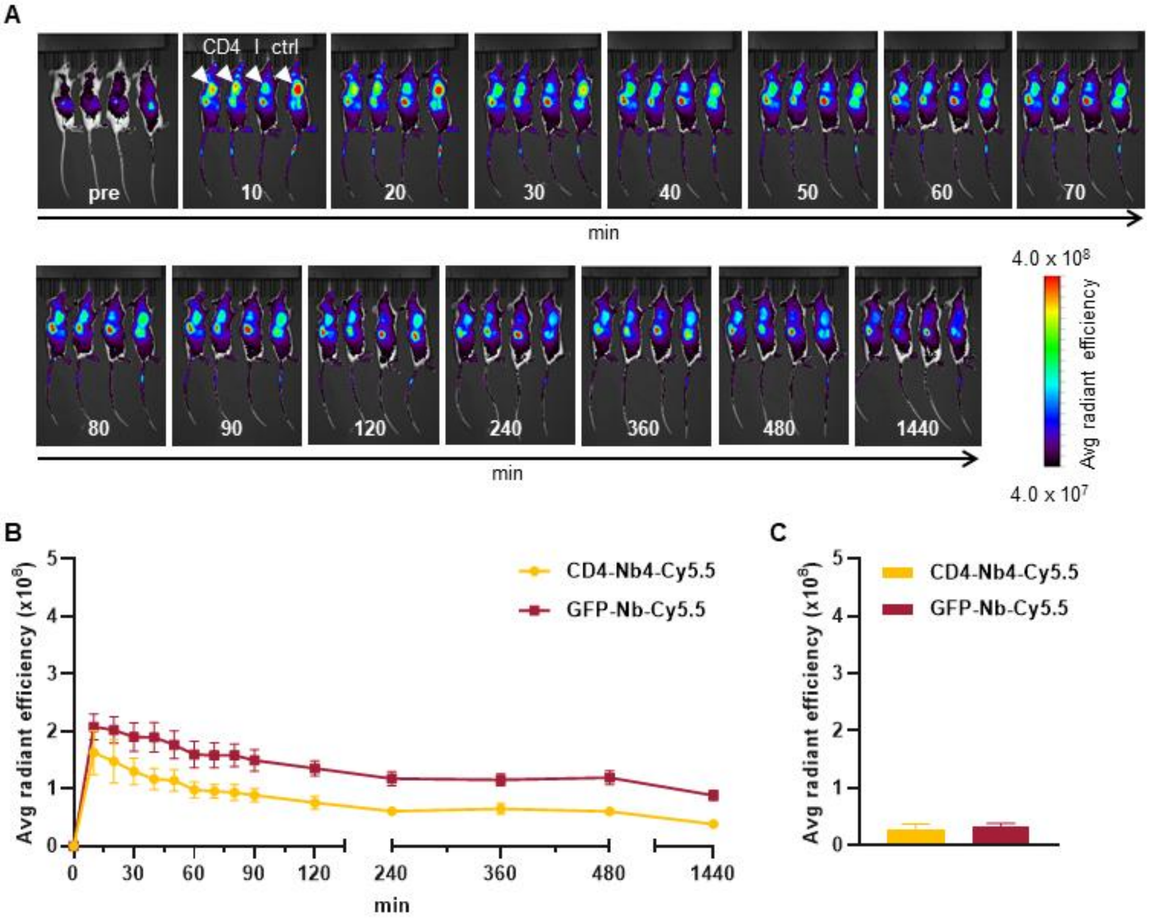

Supplementary Figure 10 *In vivo* optical imaging (OI) of low-affinity binding CD4-Nb4-Cy5.5. 5 µg of CD4-Nb4-Cy5.5-or GFP-Nb-Cy5.5 were administered *i.v.* to *subcutaneously* human CD4$^+$ HPB-ALL-bearing NSG mice and tumor bio distribution was monitored by repetitive OI measurements over the course of 24 h. (**A**) Representative images of each measurement time point of 4 mice injected either with CD4-Nb4-Cy5.5 (left, CD4) or GFP-Nb-Cy5.5 (right, ctrl). White arrows indicate the tumor localization at the right upper flank. (**B**) Quantification of the fluorescence signal from the tumors (n = 4 per group, arithmetic mean of the average radiant efficiency ± SEM). (**C**) After the last imaging time point, tumors were explanted for *ex vivo* OI, demonstrating similar accumulation of CD4-Nb4-Cy5.5 and GFP-Nb-Cy5.5 (n = 2 per group, arithmetic mean ± SEM)

Supplementary Figure 11

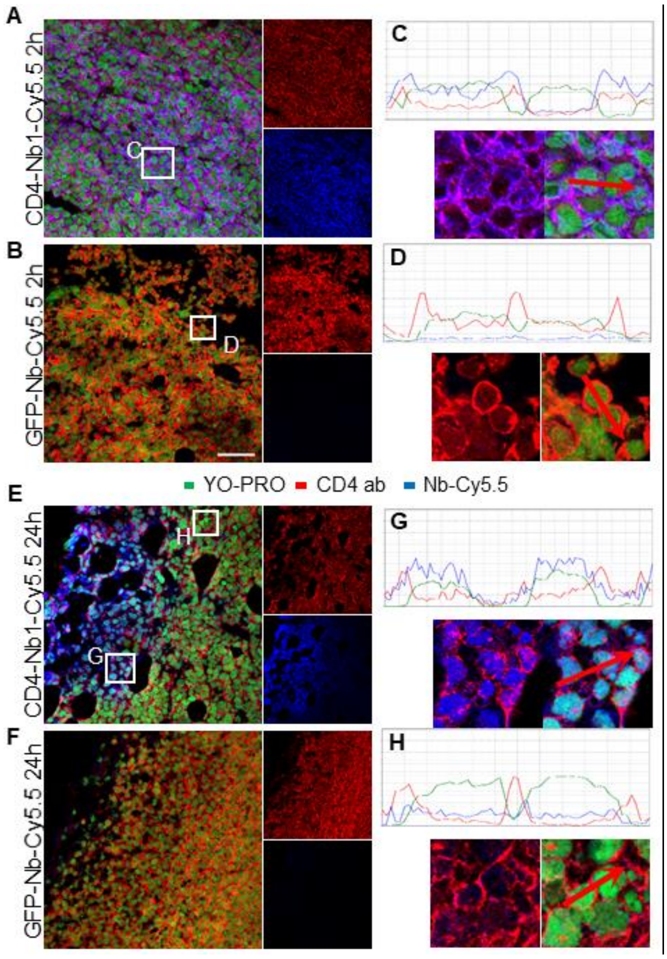

Supplementary Figure 11 Immunofluorescence staining of *ex vivo* HPB-ALL tumors. Cryosections from *ex vivo* HPB-ALL tumors were imaged for bound Nb (blue) from the systemic injection in xenografted mice and co-stained with CD4-specific antibody (red) and nuclear YO-PRO staining (green). Image overlay of CD4-Nb1 (**A**) or control GFP-Nb (**B**) with CD4 antibody fluorescence after 2 hours of Nb injection. (**C**, **D**) Line-scan quantification of indicated image sections from A and B. (**E**, **F**) same as A and B after 24 h of systemic Nb injection. (**G**, **H**) Line-scan analysis of indicated image sections from E of strongly stained area (G) or weakly stained area (H).

Supplementary Figure 12

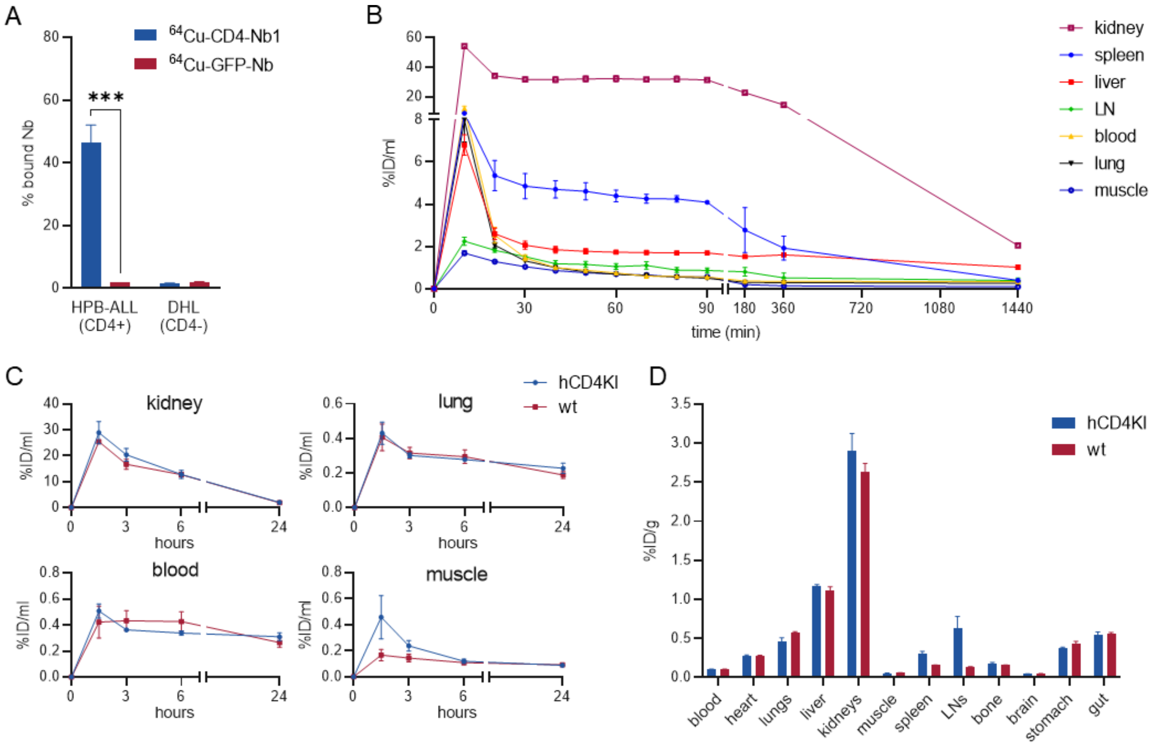

Supplementary Figure 12 ^{64}Cu-CD4-Nb1 specifically accumulates in CD4^{+} T cell-rich organs. (**A**) *in vitro* binding of ^{64}Cu-CD4-Nb1 or ^{64}Cu-GFP-Nb to excess of CD4^{+} HPB-ALL or CD4^{-} DHL control cells analyzed by γ-counting (triplicates, arithmetic mean± SD, unpaired t-test, (***) $p<0.001$)). (**B**) Dynamic *in vivo* biodistribution of ^{64}Cu-CD4-Nb1 in 2 hCD4KI mice by PET/MR. (**C**) Dynamic uptake quantification of ^{64}Cu-CD4-Nb1 in non-T cell rich organs over 24 h (n = 3 per group). (**D**) *Ex vivo* organ biodistribution analyzed by γ-counting.

Supplementary Methods

Name	Sequence 5´- 3´	purpose
CALL001	GTCCTGGCTGCTCTTCTACAAGG	Nb library generation
CALL002	GGTACGTGCTGTTGAACTGTTCC	Nb library generation
FR1-1	CAT GGC NSA NGT GCA GCT GGT GGA NTC NGG NGG	Nb library generation
FR1-2	CAT GGC NSA NGT GCA GCT GCA GGA NTC NGG NGG	Nb library generation
FR1-3	CAT GGC NSA NGT GCA GCT GGT GGA NAG YGG NGG	Nb library generation
FR1-4	CAT GGC NSA NGT GCA GCT GCA GGA NAG YGG NGG	Nb library generation
FR1-ext1	GTAGGCCCAGCCGGCCATGGCNSANGTGCAGCTGGTGG	Nb library generation
FR1-ext2	GTAGGCCCAGCCGGCCATGGCNSANGTGCAGCTGCAGGA	Nb library generation
FR4-1	GAT GCG GCC GCN GAN GAN ACG GTG ACC NGN RYN CC	Nb library generation
FR4-2	GAT GCG GCC GCN GAN GAN ACG GTG ACC NGN GAN CC	Nb library generation
FR4-3	GAT GCG GCC GCN GAN GAN ACG GTG ACC NGR CTN CC	Nb library generation
FR4-4	GAT GCG GCC GCR CTN GAN ACG GTG ACC NGN RYN CC	Nb library generation
FR4-5	GAT GCG GCC GCR CTN GAN ACG GTG ACC NGN GAN CC	Nb library generation
FR4-6	GAT GCG GCC GCR CTN GAN ACG GTG ACC NGR CTN CC	Nb library generation
NGS fwd	ACACTCTTTCCCTACACGACGCTCTTCCGATCTGGATTGTTATTACTCGCGGCC	library PCR for NGS
NGS rev	GACTGGAGTTCAGACGTGTGCTCTTCCGATCTGATCAGCTTCTGTTCTGCGGC	library PCR for NGS
hCD4 fwd	GGAGGATCCACAATGAACCGGGGAGTCCCTT	hCD4 expression
hCD4 rev	GGTCTCGAGTCAAATGGGGCTACATGTCTTCTGA	hCD4 expression
bsd fwd	ATACCCGGGGCCACCATGGCCAAGCCTTTGTCTC	hCD4 expression
bsd rev	TATGCGCGCTTAGCCCTCCCACACATAACCAG	hCD4 expression
ΔD1 fwd	GCAGTCTCTCACTGGAGCAGCGCGTTCGGATTGACTGCCAACTCTGACACC	CD4_ΔD1 expression
ΔD1 rev	GCGCACGCGATCAGGCATTCCCTGAGTGGCTGCTGGGAGG	CD4_ΔD1 expression
ΔD1ΔD2 fwd	GCAGTCTCTCACTGGAGCAGCGCTTTCCAGAAGGCCTCCAGCATAG	CD4_ΔD1ΔD2 expr.
ΔD1ΔD2 rev	GCGCACGCGATCAGGCATTCCCTGAGTGGCTGCTGGGAGG	CD4_ΔD1ΔD2 expr.
ΔD3ΔD4 fwd	CCCACATGGTCCACCCCG	CD4_ΔD3ΔD4 expr.
ΔD3ΔD4 rev	AGCTAGCACCACGATGTCTATTTTG	CD4_ΔD3ΔD4 expr.
CD4-D1-4 f	ATACGTCTCAACTCTAAGAAAGTGGTGCTGGGCAAAAAAGG	CD4-D1-4 production
CD4-D1-4 r	TATGAATTCAGTGGTGATGGTGGTGGTGGGGCAGAACCTTGATGTTGGATTCC	CD4-D1-4 production

Table S1: primers used in this study

**Cytokine**	**indicative for**
Interleukin 1 β (IL-1β)	proinflammatory
Interleukin 1 receptor antagonist (IL-1RA)	antiinflammatory
Interleukin 4 (IL-4)	antiinflammatory
Interleukin 6 (IL-6)	pro-/antiinflammatory
Interleukin 8 (IL-8)	proinflammatory
Interleukin 10 (IL-10)	antiinflammatory
Interleukin 12 p70 (IL-12p70)	proinflammatory
Interleukin 13 (IL-13)	antiinflammatory
Granulocyte-macrophage colony-stimulating factor (GM-CSF)	pro-/antiinflammatory
Interferon γ (IFNγ)	proinflammatory
Monocyte chemoattractant protein 1 (MCP-1)	proinflammatory
Macrophage inflammatory protein 1 β (MIP-1 β)	proinflammatory
Tumor necrosis factor α (TNF-α)	proinflammatory
Vascular endothelial growth factor (VEGF)	wound healing factor

Table S2: cytokines analyzed in this study

**Table HDX summary**	
State	CD4 & CD4 bound by Nb1; Nb2; Nb3
HDX reaction details	1 x PBS pH 7.4, 25 °C, 90% D_2O
Time points	5 & 50 min
Av. back exchange (Synthetic peptides)	24%
Digest conditions	2 min in an water ice-bath, 30 µl pepsin beads
Number of identified Peptides /Sequence coverage	116 / 88%
Average peptide length / average redundancy	14.6 (Std. Dev. 8.3) / 4.6
Technical replicates (triplicate)	min 2 of 3 peptides per time point, in both states
Determined ΔHX threshold for each time point	0.25 – 0.27 Da
Significant differences in HDX	Students t-distribution on 95% confidence level

Table S3: HDX-MS Summary
